# Lipoarabinomannan and related glycoconjugates: structure, biogenesis and role in *Mycobacterium tuberculosis* physiology and host–pathogen interaction

**DOI:** 10.1111/j.1574-6976.2011.00276.x

**Published:** 2011-05-31

**Authors:** Arun K Mishra, Nicole N Driessen, Ben J Appelmelk, Gurdyal S Besra

**Affiliations:** 1 School of Biosciences, University of BirminghamEdgbaston, Birmingham, UK; 2 Department of Medical Microbiology and Infection Control, VU University Medical CenterAmsterdam, The Netherlands

**Keywords:** bacterial, polysaccharides, cell wall, biosynthesis, glycosyltransferases, drug-targets

## Abstract

Approximately one third of the world's population is infected with *Mycobacterium tuberculosis*, the causative agent of tuberculosis. This bacterium has an unusual lipid-rich cell wall containing a vast repertoire of antigens, providing a hydrophobic impermeable barrier against chemical drugs, thus representing an attractive target for vaccine and drug development. Apart from the mycolyl–arabinogalactan–peptidoglycan complex, mycobacteria possess several immunomodulatory constituents, notably lipomannan and lipoarabinomannan. The availability of whole-genome sequences of *M. tuberculosis* and related bacilli over the past decade has led to the identification and functional characterization of various enzymes and the potential drug targets involved in the biosynthesis of these glycoconjugates. Both lipomannan and lipoarabinomannan possess highly variable chemical structures, which interact with different receptors of the immune system during host–pathogen interactions, such as Toll-like receptors-2 and C-type lectins. Recently, the availability of mutants defective in the synthesis of these glycoconjugates in mycobacteria and the closely related bacterium, *Corynebacterium glutamicum*, has paved the way for host–pathogen interaction studies, as well as, providing attenuated strains of mycobacteria for the development of new vaccine candidates. This review provides a comprehensive account of the structure, biosynthesis and immunomodulatory properties of these important glycoconjugates.

## Introduction

Tuberculosis (TB) is a major cause of death worldwide, with approximately 9 million cases and 1.7 million deaths registered in 2008 (WHO, 2009). To compound this situation, 50 000 cases were reported as multidrug-resistant tuberculosis (MDR-TB) and 55 countries globally had reported at least one case of extensively drug-resistant tuberculosis (XDR-TB) (WHO, 2009). *Mycobacterium tuberculosis* is the causative agent of tuberculosis. It has an unusual lipid-rich cell wall that is unique to the order *Actinomycetes*, including the genera *Mycobacterium*, *Rhodococcus*, *Corynebacterium* and *Nocardia* (Brennan & Nikaido, 1995). The mycobacterial cell wall is composed of a mycolyl–arabinogalactan–peptidoglycan (mAGP) complex (Daffé*et al.*, 1990; McNeil *et al.*, 1990, 1991; Besra *et al.*, 1995; Brennan, 2003; Dover *et al.*, 2004), of which the mycolic acids and extractable lipids form the mycobacterial outer membrane (Hoffmann *et al.*, 2008). The mycolic acid layer provides a hydrophobic mesh for intercalating additional complex lipids, resulting in a highly impermeable barrier for the penetration of antimicrobial drugs, such as penicillins (Amberson *et al.*, 1931; Minnikin *et al.*, 2002;). Other cell wall-associated lipids, such as phosphatidyl-*myo*-inositol mannosides (PIMs) and lipoglycans, termed lipomannan (LM) and lipoarabinomannan (LAM), are also found in the cell wall (Hill & Ballou, 1966; Brennan & Ballou, 1967, 1968a; Brennan & Nikaido, 1995; Besra *et al.*, 1997; Morita *et al.*, 2004). In addition to their physiological function, these complex glycoconjugates play a key role in modulating the host response during infection. PIMs, lipomannan and lipoarabinomannan all display several immunomodulatory properties by interaction with different receptors of the immune system. While lipomannan is mainly associated with Toll-like receptors (TLR) signaling, the higher-order PIMs and mannose-capped lipoarabinomannan (Man-LAM) are recognized by the C-type lectins, such as dendritic cell-specific intercellular adhesion molecule-3 (ICAM-3) grabbing nonintegrin (DC-SIGN) and the macrophage mannose receptor (MMR) (Schlesinger *et al.*, 1994; Chatterjee & Khoo, 1998; Nigou *et al.*, 2002; Geijtenbeek *et al.*, 2003; Maeda *et al.*, 2003;).

Because of the advent of MDR and XDR strains of *M. tuberculosis* (Sreevatsan *et al.*, 1997; Telenti *et al.*, 1997; Heymann *et al.*, 1998; Chan & Iseman, 2008; Wright *et al.*, 2009;), there is an urgent need to identify novel drug targets and the development of active compounds. In this respect, the biosynthetic machinery of the mycobacterial cell wall, which is the site of action of many front-line tuberculosis drugs, represents an attractive drug target (Bhatt *et al.*, 2007; Bhowruth *et al.*, 2007; Brennan & Crick, 2007; Dover *et al.*, 2008;). Furthermore, a complete investigation of the roles of PIMs, lipomannan and lipoarabinomannan in mycobacterial pathogenicity requires mutants defective in their respective biosynthetic pathways. The availability of complete genome sequences of several mycobacteria and related actinomycetes and the development of novel tools for genetic manipulation have opened up the possibilities to achieve this (Cole *et al.*, 1998).

Herein, we report recent advances in the biogenesis of lipoarabinomannan and related glycoconjugates, followed by a comprehensive analysis of their role in host–pathogen interactions. Furthermore, we review the localization and trafficking of these immunomodulatory lipoglycans and discuss recent findings concerning the role of CD1, TLR, DC-SIGN and MMR in *M. tuberculosis* infection.

## Part I – Structure and biogenesis of PIMs, lipomannan and lipoarabinomannan

### Structural features of PIMs, lipomannan and lipoarabinomannan

**Fig. 1:**
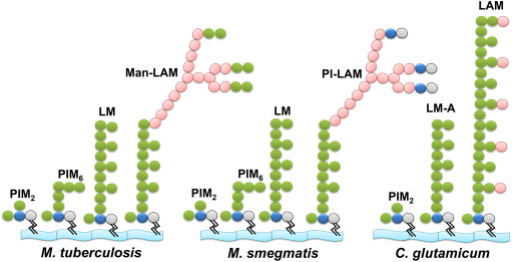
Lipoarabinomannan and related glycoconjugates found on the cell wall of *Mycobacterium tuberculosis*, *Mycobacterium smegmatis* and *Corynebacterium glutamicum*. Biochemical analysis of the mycobacterial cell wall suggests that different acylated variants of di- and hexa-mannosylated PIMs, Ac_1_/Ac_2_PIM_2_ and PIM_6_, and the higher glycosylated polymers lipomannan and lipoarabinomannan accumulate in the cell wall. However, in *C. glutamicum*, only PIM_2_, two types of lipomannan (LM-A and LM-B, Tatituri *et al.*, 2007a; Mishra *et al.*, 2008b;) and singular Ara*f* capped lipoarabinomannan are present on the cell wall. For the purpose of simplicity, only diacylated forms of these glycoconjugates and LM-A, i.e. MPI anchored lipomannan, are shown. In these glycoconjugates, phosphatidyl-*myo*-inositol (phosphate in gray and inositol in blue) acts as an anchor to the plasma membrane and further glycosylated by Man*p* (green) and Ara*f* (pink) sugars yielding different forms of PIMs, lipomannan and lipoarabinomannan that are species specific. In *M. tuberculosis* and other pathogenic mycobacteria, lipoarabinomannan is capped by mono, -di or -tri α(1→2)-Man*p* units, resulting in Man-LAM, while in nonpathogenic *M. smegmatis*, lipoarabinomannan is terminated by phospho inositol, yielding PI-LAM.

**Fig. 2:**
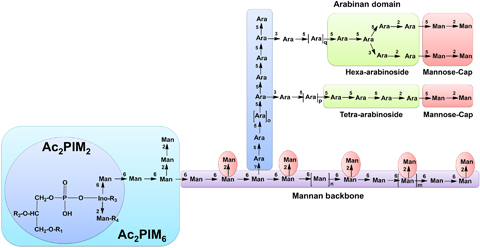
Schematic structures of lipoarabinomannan and related glycoconjugates. As described in the text, PI acts as an anchor around which PIMs, lipomannan and lipoarabinomannan are built. PI is glycosylated at the 2-OH and 6-OH positions of inositol by Man*p* residues, and acylated at position 3 of *myo*-inositol and position 6 of the Man*p* unit linked at O-2 of *myo*-inositol in Ac_2_PIM_2_ (See the inset in light blue color). Man*p* at the 6-OH position of inositol is linked to further three and two residues of α(1→6)-Man*p* and α(1→2)-Man*p*, respectively, in Ac_2_PIM_6_ (see the inset in light indigo color). In lipomannan and the mannan backbone of lipoarabinomannan, PIM_2_ is linked to another 17–19 residues of Man*p* in the α(1→6) direction and 7–9 singular branched α(1→2)-Man*p* units. Mature lipomannan is further linked via an unknown linkage to an arabinan domain made up of approximately 70 Ara*f* residues. The majority of the arabinan domain consists of a linear α(1→5)-Ara*f* polymer branched at certain positions, with α(3→5)-Ara*f* residues towards its nonreducing end resulting in a linear tetra-arabinoside or/and branched hexa-arabinoside domain, which in turn is terminated by β(1→2)-Ara*f* and capped by α(1→2)-Man*p* units. Here R_1_, R_2_, R_3_ and R_4_ show different acyl groups found at different locations in the MPI anchor, and n, m, o, p and q represent different degrees of species-specific glycosylation in lipomannan and lipoarabinomannan.

The majority of bacteria from suborder *Corynebacterineae*, including *Corynebacterium diphtheriae*, *Corynebacterium glutamicum*, pathogenic *M. tuberculosis* complex and nonpathogenic *Mycobacterium smegmatis*, possess the amphipathic lipoglycans, lipoarabinomannan and other related glycoconjugates, lipomannan and PIMs (Fig. 1). All *Mycobacterium* species possess two forms of acylated PIMs, tri- and tetra-acylated (Ac_1_- and Ac_2_-) phospho-*myo*-inositol-dimannoside (PIM_2_) and tri- and tetra-acylated phospho-*myo*-inositol-hexamannoside (Ac_1_/Ac_2_PIM_6_) (we have used Ac_1_/Ac_2_PIM_*x*_ for two different acylated versions of PIMs throughout the text, and PIM as a synonym where the acylation state is not clear), and different acylated versions of lipomannan and lipoarabinomannan (Khoo *et al.*, 1995a), which are believed to be noncovalently attached to the cell membrane via a lipid anchor (Fig. 2) (Hunter & Brennan, 1990).

#### Structure of PIMs

PIMs are categorized as glycolipids composed of fatty acids attached to a glycerol unit, linked by a phosphodiester moiety to *myo*-inositol (Vilkas & Lederer, 1956; Ballou *et al.*, 1963;) (see Fig. 2 for Ac_2_PIM_2_ and Ac_2_PIM_6_). This phosphatidyl-*myo*-inositol (PI) is based on an *sn*-glycero-3-phos-pho-(1-d-*myo*-inositol) unit and is further substituted at the O-2 and O-6 positions of *myo*-inositol with α-d-mannopyranosyl (Man*p*) units in case of PIM_2_, resulting in a mannosyl phosphate inositol (MPI) anchor, a derivative of the typical glycosyl phosphate inositol anchor, found in Eukaryotes (Lee & Ballou, 1964; Chatterjee *et al.*, 1992a; Severn *et al.*, 1998;).

The MPI anchor is heterogeneous, with variations occurring within the number, location and nature of the fatty acids. There are four potential sites of acylation within the MPI anchor, with different fatty acids at 1-OH and 2-OH of the glycerol unit in the anchor, 3-OH of *myo*-inositol and the 6-OH of the Man*p* residue linked at the O-2 position of *myo*-inositol (see R_1_, R_2_, R_3_ and R_4_ in Fig. 2) (Khoo *et al.*, 1995a; Nigou *et al.*, 2003;). Two different acylated forms of PIMs accumulate in the cell wall of mycobacteria, one with an acyl group at either the 3-OH of *myo*-inositol or the 6-OH of the Man*p* residue linked at the O-2 position of *myo*-inositol, Ac_1_PIM_*x*_, and secondly with an acyl group at both positions, Ac_2_PIM_*x*_. In mycobacteria, palmitic (C_16_) and tuberculostearic (10-methyl-octadecanoic, C_19_) acids are predominant, while myristic (C_14_) and octadecenoic acids (C_18 : 1_) are also found in significant amounts, with traces of stearic (C_18_), hexadecenoic (C_16 : 1_) and heptadecanoic acids (C_17_) (Ballou & Lee, 1964; Lee & Ballou, 1964; Gilleron *et al.*, 2003; Nigou *et al.*, 2003;). Furthermore, it was suggested that the 6-OH position of the O-2 mannose attached to the inositol of PIM_2_ is substituted by a C_16_ fatty acyl-substituent, which is also present in lipomannan and lipoarabinomannan from *M. tuberculosis* and *Mycobacterium leprae* (Khoo *et al.*, 1995a).

**Fig. 4:**
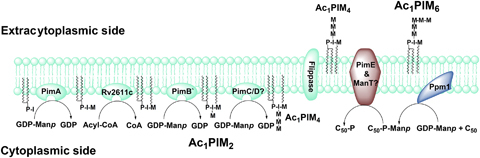
Overview of PIM biosynthesis in *Mycobacterium tuberculosis*. On the cytosolic side of the plasma membrane, PI is glycosylated by PimA, PimB' and an acyltransferase to form Ac_1_PIM_2_, which is further mannosylated by PimC and/or PimD? to form Ac_1_PIM_4_, an intermediate in Ac_1_PIM_6_ and lipomannan biosynthesis. Ac_1_PIM_4_ is probably transported across the plasma membrane by unidentified flippases and further mannosylated by α(1→2) mannopyranosyltransferases, PimE and/or another unidentified enzyme to form Ac_1_PIM_6_. For simplicity, only triacylated versions of PIMs are shown.

Acylated forms of PIM_2_ serve as substrates for the synthesis of higher-order PIMs, such as Ac_1_/Ac_2_PIM_6_ (Figs 2 and 4). Studies with a crude cell extract of *M. tuberculosis* and *Mycobacterium phlei* identified PIM_6_, which is a pentamannoside attached to the position O-6 of the *myo*-inositol of PI of PIM_1_, Man*p*-α(1→2)-Man*p*-α(1→2)-Man*p*-α(1→6)-Man*p*-α(1→6)-Man*p*-α(1→.) (Lee & Ballou, 1965), which was later verified by others (Chatterjee *et al.*, 1992a; Severn *et al.*, 1998;) (Fig. 2). A biosynthetic relationship between PIM_1_ and PIM_2_ was also suggested, which involves a stepwise glycosylation of PI, first at the O-2 position and then at the O-6 position of the inositol ring (Ballou & Lee, 1964; Chatterjee *et al.*, 1992a;). It was also suggested that this acylated version of PIM_2_ i.e. Ac_1_PIM_2_ is both a metabolic end-product and an intermediate in Ac_1_PIM_6_, lipomannan and lipoarabinomannan biosynthesis (Chatterjee *et al.*, 1992a; Khoo *et al.*, 1995a; Besra *et al.*, 1997;).

More recently, the presence of a glucuronic acid diacyl-glycerol-based glycolipid, Man*p*-α(1→4)-d-glucopyranosyluronic acid-diacyl glycerol, ManGlcAGroAc_2_, and a novel lipomannan variant (Cg-LM-B) were reported in *C. glutamicum*, in addition to the MPI anchored-LM (Cg-LM-A) and lipoarabinomannan, and to date, these glycoconjugate variants have not been identified in mycobacteria (Tatituri *et al.*, 2007a; Lea-Smith *et al.*, 2008; Mishra *et al.*, 2008b, 2009). However, Rv0557 [MgtA] of *M. tuberculosis* was shown to have the ability to synthesize these novel lipids and lipoglycans *in vitro* and *in vivo* (Tatituri *et al.*, 2007a; Mishra *et al.*, 2009;), and the majority of the genus *Mycobacterium* possesses an ortholog of MgtA. Therefore, theoretically, the possibility remains for the identification of glucuronic acid-anchor-based glycolipids in mycobacteria in addition to MPI anchor-based ones.

#### Structure of lipomannan and lipoarabinomannan

In 1930, Masucci and colleagues isolated a polysaccharide containing d-arabinofuranose (Ara*f*) and Man*p* with high serological activity from mycobacteria (Masucci *et al.*, 1930), which was also identified in *Mycobacterium bovis* bacilli Calmette-Guérin (BCG) (Chargaff & Schaefer, 1935), and separated using electrophoresis (Seibert & Watson, 1941). Later, one of the polysaccharides was identified as arabinogalactan (AG), the basic constituent of the mAGP complex (Misaki & Yukawa, 1966), and the other as a pool of immunologically active lipoarabinomannan and inactive lipomannan (Misaki *et al.*, 1977), both sharing a similar mannan core (Hunter *et al.*, 1986; Hunter & Brennan, 1990; Chatterjee *et al.*, 1992a;) (Fig. 2). Further studies identified Ac_1_/Ac_2_PIM_2_ as the attachment point (Hunter & Brennan, 1990) for the synthesis of the α(1→6)-mannan backbone of lipomannan and lipoarabinomannan, which is composed of around 21–34 residues of α(1→6)-Man*p* and decorated by singular 5–10 units of α(1→2)-Man*p*, resulting in the formation of lipomannan (Chatterjee *et al.*, 1992a; Kaur *et al.*, 2008;). The mannan core is further elaborated by the addition of an arabinan domain consisting of approximately 55–70 Ara*f* residues in a linear α(1→5)-d-Ara*f* fashion with 3,5-α-d-Ara*f* branches (Kaur *et al.*, 2008; Birch *et al.*, 2010;). The arabinan domain is highly branched and conserved with two types of chain arrangements. Firstly, linear tetra-arabinoside (Ara-4) of the structure β-d-Ara*f*(1→2)-α-d-Ara*f*(1→5)-α-d-Ara*f*(1→5)-α-d-Ara*f*, and secondly, branched hexa-arabinoside (Ara-6) motifs with the structure [β-d-Ara*f*(1→2)-α-d-Ara*f*]_2_-3,5-α-d-Ara*f*(1→5)-α-d-Ara*f* (Chatterjee *et al.*, 1991; Chatterjee *et al.*, 1993;). In both cases, the nonreducing end is characterized by the disaccharide unit, Ara*f*-β(1→2)-Ara*f-*α(1→.) (Fig. 2) (Chatterjee *et al.*, 1991, 1993; McNeil *et al.*, 1994).

The arabinan termini of lipoarabinomannan from the Erdman strain of *M. tuberculosis* were shown to be capped with Man*p* residues and it was established that the tetra-/hexa-arabinofuranoside unit was further extended by mono, di- and tri-α(1→2)-d-Man*p* saccharide units (Fig. 2) (Chatterjee *et al.*, 1992b, 1993; Venisse *et al.*, 1993). The number of mannose caps is species specific, with *M. tuberculosis* H37Rv and *M. bovis* BCG Man-LAM equally capped with around seven residues *per* molecule of lipoarabinomannan (Khoo *et al.*, 1995b; Nigou *et al.*, 2003;). Surprisingly, lipoarabinomannan from a fast-growing *Mycobacterium* species is devoid of any mannose cap (Chatterjee *et al.*, 1992b) and, in turn, a novel phosphoinositol capping motif was identified from *M. smegmatis* strains ATCC 14468 and mc^2^155 (PI-LAM) (Fig. 1) (Khoo *et al.*, 1995b) and the absence of a capping motif in lipoarabinomannan from *Mycobacterium chelonae*, AraLAM (Guérardel *et al.*, 2002).

#### Further chemical modifications of lipoarabinomannan

In its physiological form, Man-LAM is found in two different fractions: parietal and cellular (Vercellone *et al.*, 1998; Gilleron *et al.*, 2000; Hoffmann *et al.*, 2008;). These fractions differ in terms of the percentage of mannose caps and acylation groups of the MPI anchor. Parietal Man-LAM possess a novel fatty acid assigned as 12-*O*-(methoxypropanoyl)-12-hydroxystearic acid, esterified at *C*-1 of the glycerol residue of PI, while cellular Man-LAMs are largely heterogeneous with palmitic and tuberculostearic acid (Nigou *et al.*, 1997). More likely, cellular lipoarabinomannan is more strongly attached to the cell wall due to higher acylation as compared with parietal lipoarabinomannan (Pitarque *et al.*, 2008). Furthermore, in different *M. bovis* BCG strains (Pasteur, Glaxo, Copenhagen and Japanese strains), the presence of succinyl groups on O-2 of the 3,5-di-α-d-Ara*f* residue of Man-LAM was also reported (Delmas *et al.*, 1997). Recently, Treumann *et al.* (2002) identified a 5-methylthiopentose substituent on the terminal Man*p* in the cap structure of Man-LAM in several strains of *M. tuberculosis*, which was later characterized as 5-deoxy-5-methylthio-xylofuranose (Turnbull *et al.*, 2004) with a d-configuration (Joe *et al.*, 2006) and linked by an α(1→4) linkage to a Man*p* residue in the mannan portion of the glycan (Guérardel *et al.*, 2003).

### Biogenesis of PIMs, lipomannan and lipoarabinomannan

#### Biosynthesis of substrates

##### GDP-Manp biosynthesis

Besides being part of glycolipids and lipoglycans, mannose is also involved in the synthesis of a number of glycosylated proteins (VanderVen *et al.*, 2005) and a few other key components, such as polymethylated polysaccharides in mycobacteria (Jackson & Brennan, 2009). These molecules are synthesized by both pathogenic and nonpathogenic species, raising the possibility of as yet undefined ‘housekeeping’ functions in these organisms. The mannose metabolism is essential for growth in *M. smegmatis* and it was suggested that apart from glycolipid and lipoglycan biosynthesis, mannose-containing molecules may also play a role in regulating septation and cell division (Patterson *et al.*, 2003).

**Fig. 3:**
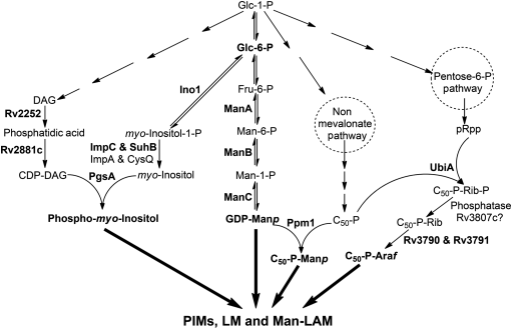
Biosynthetic pathways of important nucleotide and lipid-linked sugar donors involved in the synthesis of PIMs, lipomannan and Man-LAM. Most of the sugars utilized by mycobacteria are derived from glycolytic intermediates or glucose as the major carbon source. The experimentally characterized enzymes have been indicated in bold. Apart from the glycolytic pathway, GDP-Man*p*, C_50_-P-Man*p*, C_50_-P-Ara*f* and PI are also derived from exogenous sources, which have not been shown here to retain simplicity.

In mycobacteria, mannose is probably obtained by two distinct pathways: firstly, by transport of extracellular mannose from the medium or the extracellular environment with the activity of a hexokinase (Kowalska *et al.*, 1980). The phosphorylated mannose, mannose-1-phosphate, is then converted into GDP-Man*p* by GDP-mannose pyrophosphorylase, ManC [Rv3264c] (Ning & Elbein, 1999; Ma *et al.*, 2001;) (Fig. 3). Secondly, in the absence of extracellular mannose, it can be derived from glucose and other sugars via the glycolytic pathway, where fructose-6-phosphate is converted to mannose-6-phosphate by an essential enzyme, phosphomannose isomerase, encoded by *manA* [Rv3255c] (Patterson *et al.*, 2003). Mannose-6-phosphate is then converted to mannose-1-phosphate by a phosphomannomutase, ManB [Rv3257c] (McCarthy *et al.*, 2005), followed by conversion into GDP-Man*p* by ManC (Ning & Elbein, 1999; Ma *et al.*, 2001;) (Fig. 3).

##### Synthesis of β-d-mannosyl-1-monophosphoryldecaprenol

GDP-Man*p* serves as an intracellular nucleotide-derived mannose donor for the synthesis of several glycolipids and mannosylated proteins by the GT-A/B superfamily of glycosyltransferases (Liu & Mushegian, 2003). However, for periplasmic biosynthetic events, a polyprenyl-phosphate-based mannose donor is required, which acts as a mannose donor for the GT-C superfamily of glycosyltransferases for the synthesis of higher PIMs, lipomannan and lipoarabinomannan. Takayama & Goldman (1970) were the first to report the presence of a C_50_-polyprenol-based mannolipid, C_50_-decaprenol-phospho-mannose (C_50_-P-Man*p*, PPM), in *M. tuberculosis* (Takayama & Goldman, 1970). Later on, another alkali-stable, C_35_-octahydroheptaprenyl-phospho-mannose, C_35_-P-Man*p*, was identified in *M. smegmatis* (Wolucka & Hoffmann, 1998). Based on similarities to the known eukaryotic dolichol monophosphomannose synthases, Rv2051c [Ppm1] from *M. tuberculosis* was identified as a polyprenol monophosphomannose synthase, PPM synthase (Gurcha *et al.*, 2002) (Figs 3 and 4). Surprisingly, Ppm1 possesses an unusual two-domain architecture in *M. tuberculosis*, of which the second domain, Mt-Ppm1/D2, is sufficient for PPM synthesis (Gurcha *et al.*, 2002; Gibson *et al.*, 2003;). However, *M. smegmatis*, *Mycobacterium avium* and *M. leprae* produce two distinct proteins, one for each of the two domains found in Mt-Ppm1, with Ms-Ppm2 and Ma-Ppm2 having a catalytic activity similar to that of domain 2 of Mt-Ppm1. Recently, a transmembrane glycosyltransferase, Rv3779, was identified and suggested to be involved in the synthesis of C_35/50_-P-Man*p* as a second PPM synthase (Scherman *et al.*, 2009). However, Skovierova *et al.* (2010) recently described the function of Rv3779 as the glycosyltransferase involved in transferring galactosamine from a polyprenyl-phospho-*N*-acetylgalactosamine to arabinogalactan in *M. tuberculosis*.

##### Origin and synthesis of decaprenyl-phospho-arabinose

Generally, in nature, d-arabinose exists in two cyclic forms: a rare pyranose-ring (Ara*p*) and the furanose-ring (Ara*f*) (Wolucka, 2008). Ara*f* forms a key component of both arabinogalactan and lipoarabinomannan in mycobacteria and the only known Ara*f* sugar donor is a lipid-linked decaprenyl-phospho-arabinose (DPA and also termed C_50_-P-Ara*f*) (Wolucka *et al.*, 1994). However, a putative role of a nucleotide-based Ara*f* donor was also suggested in the addition of the single terminal d-Ara*f* residues of lipoarabinomannan in *C. glutamicum* (Tatituri *et al.*, 2007b). The majority of DPA synthesized in mycobacteria comes from the pentose phosphate pathway (Marks, 1956). A transketolase, Rv1449, links the glycolytic and pentose phosphate pathway to produce ribose-5-phosphate (Wolucka, 2008). Alternatively, ribose-5-phosphate isomerase [Rv2465] isomerizes d-ribulose-5-phosphate into ribose-5-phosphate (Roos *et al.*, 2004; Roos *et al.*, 2005;). Furthermore, Rv1017c, a ribose-5-phosphate diphosphokinase (PrsA), converts ribose 5-phosphate into 5-phosphoribosyl-α-1-pyrophosphate (pRpp) (Alderwick *et al.*, 2010), which is dephosphorylated by a phosphatase as the first committed step in decapolyprenol-phosphoribose (DPR and also termed C_50_-P-Rib) and DPA biosynthesis (Mikusová*et al.*, 2005) (Fig. 3). In the genome of *M. tuberculosis*, an unknown poly-(A)-polymerase2 (PAP2)-superfamily phospholipid phosphatase [Rv3807c] exists that is present in the arabinogalactan biosynthetic cluster (Rv3779-Rv3809c) and next to Rv3806c, UbiA (Huang *et al.*, 2005), which may be responsible for pRpp phosphatase activity. Furthermore, the deletion of the Rv3807c homolog in *C. glutamicum* remains unsuccessful, suggesting it as a prime candidate (L. Eggeling & G.S. Besra, unpublished data).

The synthesis of DPA and DPR from pRpp was shown experimentally and it was concluded that DPA is formed from pRpp via a two-step pathway, with an additional epimerization step that converts DPR to DPA (Scherman *et al.*, 1996; Mikusová*et al.*, 2005;) (Fig. 3). Recently, a 5-phospho-α-d-ribose-1-diphosphate:decaprenyl-phosphate 5-phospho-ribosyltransferase, UbiA [Rv3806c], was identified in *M. tuberculosis* (Huang *et al.*, 2005). The deletion of *ubiA* in *C. glutamicum* produced a mutant that possessed a galactan core consisting of alternating β(1→5)-galactofuranose (Gal*f*) and β(1→6)-Gal*f* residues and completely devoid of arabinan and cell-wall-bound corynomycolic acids, confirming its role in the synthesis of DPR and DPA biosynthesis in *Corynebacterineae* (Alderwick *et al.*, 2005; Alderwick *et al.*, 2006a;). More recently, Mikusová*et al.* (2005) identified an epimerase, which is involved in the epimerization of DPR to DPA. It was established that the 2-OH of ribose is oxidized to decaprenylphosphoryl-2-keto-β-d-*erythro*-pentofuranose, which is then reduced to form DPA. These activities are encoded by Rv3790 and Rv3791, respectively, and the simultaneous expression of both is required for complete activity of the epimerase reaction (Mikusová*et al.*, 2005) (Fig. 3). Interestingly, Rv3790 has been shown to be a target of benzothiazinones, potential tuberculosis drugs (Christophe *et al.*, 2009; Makarov *et al.*, 2009;).

##### Synthesis of phosphatidyl-myo-inositol

Inositol is an essential metabolite in *Mycobacterium* (Kataoka & Nojima, 1967), *Corynebacterium* (Brennan & Lehane, 1971), *Nocardia* (Yano *et al.*, 1969), *Micromonospora* (Tabaud *et al.*, 1971) and *Propionibacterium* (Brennan & Ballou, 1968b). In mycobacteria, inositol is essential for growth and derived directly via glycolysis (Jackson *et al.*, 2000). Glucose-6-phosphate is cyclized by an inositol-1-phosphate synthase, Ino1 [Rv0046c] (Bachhawat & Mande, 1999; Movahedzadeh *et al.*, 2004;), into *myo*-inositol-1-phosphate, followed by its dephosphorylation utilizing an inositol monophosphatase (IMP) (Fig. 3). On the basis of homology, the *M. tuberculosis* genome shows four ORFs encoding putative proteins with an IMP domain: Rv1604 (ImpA), Rv2701c (SuhB), Rv2131c (CysQ) and Rv3137 (ImpC). Out of these, *impC* was found to be essential for mycobacterial growth, and it was suggested that *impA*, *suhB* and *cysQ* may make a minor contribution towards inositol biosynthesis (Movahedzadeh *et al.* 2010) as suggested by the *in vitro* IMP activity of SuhB (Fig. 3) (Parish *et al.*, 1997; Nigou & Besra, 2002a; Brown *et al.*, 2007;).

The first step in the production of many phospholipids, including PI, is the phosphorylation of diacylglycerol (DAG) by a diacylglycerol kinase [Rv2252] to form phosphatidic acid (Owens *et al.*, 2006). Phosphatidic acid is then activated by CTP to form CDP-DAG by a CDP-DAG synthase [Rv2881c], a homolog of which has been characterized in *M. smegmatis* (Nigou & Besra, 2002b). Furthermore, it was shown that a cell wall fraction (Percoll-60, P_60_) from *M. smegmatis* is able to synthesize P-[^3^H]-I in the presence of the exogenous substrate, CDP-dipalmitoyl-DAG, concluding that *myo*-inositol reacts with CDP-DAG and forms PI (Salman *et al.*, 1999). Recently, the gene encoding the PgsA [Rv2612c] has been identified and shown to be essential in *M. tuberculosis* (Jackson *et al.*, 2000) (Fig. 3).

#### Overview of PIM biosynthesis

The current model of mycobacterial PIM biosynthesis supported by biochemical and genetic studies follows a linear pathway from PI→PIM_2_→PIM_4_→PIM_6_ (Fig. 4) (Chatterjee *et al.*, 1992a; Besra & Brennan, 1997; Morita *et al.*, 2004, 2006). Glycosylation of PI by different α-mannopyranosyltransferases, PimA, PimB', PimC, unidentified PimD?, PimE, unidentified PimF? and acylation by acyltransferase(s), results in the synthesis of Ac_1_/Ac_2_PIMs (Kordulakova *et al.*, 2002, 2003; Kremer *et al.*, 2002; Morita *et al.*, 2006; Lea-Smith *et al.*, 2008; Guerin *et al.*, 2009; Mishra *et al.*, 2009), out of which Ac_1_/Ac_2_PIM_2_ and Ac_1_/Ac_2_PIM_6_ accumulate onto the mycobacterial cell wall (Figs 1 and 4).

##### Conversion of PI into Ac_1_PIM_1_

The enzymes involved in the synthesis of early PIMs are encoded by a conserved cluster of six ORFs in an operon, which is found in all members of *Corynebacterineae* (Cole & Barrell, 1998; Cole *et al.*, 1998;). The first ORF of this cluster, Rv2614c, encodes a protein with an aminoacyl-tRNA synthase class-II motif and is similar to *Escherichia coli* threonyl-*t-*RNA synthases. The second ORF, Rv2613c, has similarity to the proteins involved in nucleotide biosynthesis, while the third ORF, Rv2612c, encodes for PgsA and the fourth ORF, Rv2611c, encodes an acyltransferase. An *M. smegmatis* Rv2611c mutant exhibited severe growth defects and accumulated nonacylated PIM_1_ and PIM_2_, suggesting its role in acylation of PIMs. Further biochemical analysis suggested that Rv2611c acylates the 6-position of Man*p* residue linked to the 2-OH position of *myo*-inositol (Kordulakova *et al.*, 2003). Very recently, the identification of an α-d-mannose-α(1→6)-phosphatidyl-*myo*-inositol-mannopyranosyltransferase, PimB', involved in the biosynthesis of Ac_1_/Ac_2_PIM_2_, shed further light on the acylation step in PIM biosynthesis. The deletion of *pimB*' in *C. glutamicum* resulted in the abrogation of Ac_1_/Ac_2_PIM_2_ and the accumulation of Ac_1_PIM_1_ (Lea-Smith *et al.*, 2008; Mishra *et al.*, 2008b;), suggesting that the first acylation step, i.e. acylation of PIM_1_ (Kordulakova *et al.*, 2003), precedes the second mannosylation step, resulting in the formation of Ac_1_PIM_2_ (Schaeffer *et al.*, 1999).

*PimA* [Rv2610c] is the fifth ORF of the operon and is essential in *M. smegmatis* (Kordulakova *et al.*, 2002). In cell-free assays with partially purified Rv2610c and/or membranes from *M. smegmatis* overexpressing PimA and GDP-[^14^C]-Man*p*, Kordulakova *et al.* (2002) identified the incorporation of radioactivity into PIM_1_ and Ac_1_PIM_1_. They deduced that Rv2610c encodes for an α-mannopyranosyltransferase and that PimA is responsible for the formation of PIM_1_ from PI and GDP-Man*p* (Kordulakova *et al.*, 2002). The crystal structure of PimA in complex with GDP-Man*p* from *M. smegmatis* shows a two-domain organization with the catalytic machinery typical of GT-B glycosyltransferases (Guerin *et al.*, 2007). The sixth ORF, Rv2609c, encodes for a putative GDP-Man*p* hydrolase containing a mutT domain (see below for further discussion).

##### Synthesis of Ac_1_PIM_2_, an important step in higher PIMs, lipomannan and lipoarabinomannan biosynthesis

Recently, Rv2188c and its homologs in *C. glutamicum* (Lea-Smith *et al.*, 2008; Mishra *et al.*, 2008b;) and *M. smegmatis* (Guerin *et al.*, 2009) were identified as PimB'. This identification augmented the confusion in the field, as another gene, Rv0557, had already been assigned the function of PimB as an α-d-mannose-α(1→6)-phosphatidyl-*myo*-inositol-mannopyranosyltransferase (Schaeffer *et al.*, 1999). This study was based on the utilization of cell-free assays using GDP-[^14^C]-Man*p*, Ac_1_PIM_1_, *M. smegmatis* membranes and/or partially purified recombinant Rv0557 (Schaeffer *et al.*, 1999). Furthermore, the disruption of Rv0557 in *M. tuberculosis* did not affect the biosynthesis of Ac_1_PIM_2_ (Torrelles *et al.*, 2009), suggesting either gene duplication or that Rv0557 performed another function in *M. tuberculosis*. Recently, Rv0557 was shown to be involved in the biosynthesis of ManGlcAGroAc_2_ and a Cg-LM-B (also see Structure of PIMs) in *C. glutamicum*, and has been suggested to have an α-mannosyl-glucopyranosyluronic acid transferase, MgtA, activity (Tatituri *et al.*, 2007a).

To solve this puzzle, involving PimB, PimB' and MgtA, and to assign the correct function to each ORF, a double mutant deficient in orthologs of Rv0557 and Rv2188c was generated in *C. glutamicum*, and subsequently, Rv0557 and Rv2188c were overexpressed in the double mutant. Consequently, the *in vivo* complementation of α-d-mannose-α(1→6)-phosphatidyl-*myo*-inositol-mannopyranosyltransferase activity was restored using plasmid-borne copies of Rv2188c resulting in the synthesis of Ac_1_PIM_2_ and the related lipoglycan in the *C. glutamicum* double mutant, while overexpression of Rv0557 resulted in the synthesis of ManGlcAGroAc_2_, suggesting that Rv0557 has an α-mannosyl-glucopyranosyluronic acid transferase activity, and therefore, Rv2188c was suggested to be Mt-PimB, while Rv0557 was renamed as Mt-MgtA (Mishra *et al.*, 2009). For consistency with the recent literature, we retain the designation PimB' for Rv2188c (and its orthologs in *M. smegmatis* and *C. glutamicum*) (Lea-Smith *et al.*, 2008; Mishra *et al.*, 2008b, 2009; Guerin *et al.*, 2009). The crystal structure of *C. glutamicum* PimB' in complex with GDP vs. GDP-Man*p* shows the selectivity of PimB' for 6-OH of the inositol moiety of PI (Batt *et al.*, 2010). Rv0557 possesses relaxed substrate specificity towards Ac_1_PIM_1_ (Schaeffer *et al.*, 1999; Mishra *et al.*, 2009;) and its deletion from *M. tuberculosis* resulted in a viable mutant with a subtle decrease in the lipomannan and lipoarabinomannan contents (Torrelles *et al.*, 2009), indicating a superficial role of Rv0557 in the biosynthesis of PIMs, lipomannan and lipoarabinomannan. In contrast, Rv2188c is essential in *M. smegmatis* (Guerin *et al.*, 2009), illustrating an example of a high-duplication event that lead to extensive functional redundancy in mycobacteria (Cole *et al.*, 1998; Tekaia *et al.*, 1999;).

More recently, the role of glycosyl hydrolases has been suggested in the regulation of glycolipid flux inside and outside the cell membrane and it was suggested that these glycosyl hydrolases work in close coordination with glycosyltransferases (Crespo *et al.*, 2010). The presence of GDP-Man*p* hydrolases in the vicinity of *pimA* and *pimB*', suggests the metabolic role of these glycosyl hydrolases in the regulation of the sugar donors and glycolipids, such as GDP-Man*p*, PPM and PIMs. In addition, the presence of putative transporters Rv2190c in *M. tuberculosis* and *NCgl2107* and *NCgl2108* in *C. glutamicum* in the vicinity of *pimB*' region suggests their role in PIM or PPM transport in *Corynebacterineae*. However, the deletion of the homolog of Rv2190c in *C. glutamicum* resulted in a viable mutant with no phenotype, suggesting gene redundancy, which is not surprising as these putative transporters are present in multicopies elsewhere in the genome (L. Eggeling & G.S. Besra, unpublished data). Future studies targeting the role of these glycosyl hydrolases and transporters may shed further light on the regulation of PIMs, lipomannan and lipoarabinomannan in mycobacteria.

##### Synthesis of higher-order PIMs

Bioinformatical analysis of the genome of *M. tuberculosis* CDC1551 has led to the identification of RvD2-ORF1 from *M. tuberculosis* CDC1551 as an Ac_1_PIM_2_:α-d-mannose-α(1→6)-phosphatidyl-*myo*-inositol-mannopyranosyltransferase, PimC, involved in the addition of Man*p* from GDP-Man*p* to the 6-OH of mannose at the nonreducing end of Ac_1_/Ac_2_PIM_2_ (Kremer *et al.*, 2002). The use of a cell-free assay containing GDP-Man*p*, amphomycin (an antibiotic that inhibits the synthesis of PPMs by inhibiting the PPM synthase) and membranes from *M. smegmatis-*overexpressing PimC led to the synthesis of Ac_1_/Ac_2_PIM_3_. However, the inactivation of *pimC* in *M. bovis* BCG did not affect the production of higher PIMs, lipomannan and lipoarabinomannan, and the fact that genes orthologous to *pimC* were found in only 22% of clinical isolates suggests the existence of redundant gene(s) or an alternate pathway that may compensate for PimC deficiency (Kremer *et al.*, 2002).

Ac_1_/Ac_2_PIM_3_ is further α(1→6) mannosylated at the nonreducing termini by an unidentified α(1→6)-mannopyranosyltransferase [PimD] or PimC itself, resulting in the formation of Ac_1_/Ac_2_PIM_4_. This step in the biosynthesis of higher PIMs, lipomannan and lipoarabinomannan, has been suggested as a key branch point towards the synthesis of Ac_1_/Ac_2_PIM_6_, lipomannan and lipoarabinomannan (Morita *et al.*, 2004; Morita *et al.*, 2006; Mishra *et al.*, 2008a;). It has been proposed that a transition occurs from glycosyltransferases, utilizing nucleotide-derived sugar substrates, characterized by the GT-A/B superfamily, to the glycosyltransferases utilizing polyprenyl-phosphate sugars, the GT-C superfamily (Liu & Mushegian, 2003), for the elongation and branching of lipomannan and lipoarabinomannan (Morita *et al.*, 2006). Rv1159 [PimE] has been identified as an α(1→2)-mannopyranosyltransferase that utilizes PPM as a substrate and adds an α(1→2)-Man*p* to Ac_1_/Ac_2_PIM_4_, resulting in the synthesis of Ac_1_/Ac_2_PIM_5_ (Fig. 4) (Morita *et al.*, 2006). However, it is not clear whether PimE is solely responsible for the synthesis of both Ac_1_/Ac_2_PIM_5_ and Ac_1_/Ac_2_PIM_6_. So far, most of the putative glycosyltransferases belonging to the GT-C family (Liu & Mushegian, 2003) in *M. tuberculosis* have been functionally characterized. That leaves us with fewer possibilities, in which either PimE or one of the other uncharacterized GT-Cs (Rv0051 and Rv0541c) (Liu & Mushegian, 2003; Berg *et al.*, 2007;) adds the second Man*p* residue onto Ac_1_/Ac_2_PIM_5_. However, an Rv0051 deletion mutant showed no phenotypic change in the cell wall glycolipid of *M. tuberculosis* (A.K. Mishra & G.S. Besra, unpublished data), leaving Rv0541c as a promising candidate.

Morita *et al.* (2005) suggested that enzymes involved in the biosynthesis of early PIM intermediates (PIM_1_ and Ac_1_PIM_1_) are localized to a membrane subdomain termed PM_f_ in the plasma membrane, while the majority of Ac_1_/Ac_2_PIM_2_ (and biosynthetic enzymes) involved in higher-order PIM (Ac_1_/Ac_2_PIM_4_ and Ac_1_/Ac_2_PIM_6_) biosynthesis are localized to a denser fraction that contains both plasma membrane and cell wall markers (PM-CW) (Morita *et al.*, 2005). On the basis of various cell-free assays, they concluded that higher PIM biosynthesis occurs in the plasma membrane rather than the PM-CW fraction, followed by their subsequent transport to the cell wall (Morita *et al.*, 2005). The relative amount of higher PIMs and lipoglycans was suggested to be regulated by a recently identified lipoprotein [LpqW] in *M. smegmatis* (Kovacevic *et al.*, 2006; Marland *et al.*, 2006;). However, the exact mechanism of PIM flux and its segregation for Ac_1_/Ac_2_PIM_6_ or lipomannan biosynthesis is unknown. Furthermore, Ac_1_/Ac_2_PIM_4_ was suggested to be a key regulatory product involved in the biosynthesis of Ac_1_/Ac_2_PIM_6_ and/or lipomannan biosynthesis (Morita *et al.*, 2004, 2006). PimE directs Ac_1_/Ac_2_PIM_4_ towards Ac_1_/Ac_2_PIM_6_ synthesis, while LpqW channels Ac_1_/Ac_2_PIM_4_ for lipomannan synthesis (Crellin *et al.*, 2008). It is speculated that Ac_1_/Ac_2_PIM_4_ is transported by a flippase or a sugar transporter across the plasma membrane, where subsequent mannosylation occurs by distinct mannopyranosyltransferases belonging to the GT-C family (Liu & Mushegian, 2003; Mishra *et al.*, 2008a;).

Recently, the role of a putative acyl transferase, Rv1565c, was suggested in the acylation of higher-order PIMs, lipomannan and lipoarabinomannan. An Rv1565c deletion mutant in *Mycobacterium marinum* showed a reduced incorporation of 1,2-[^14^C]-acetate into the PIMs, lipomannan and lipoarabinomannan as compared with the wild type. Furthermore, lipoarabinomannan from the mutant lacks mannose caps and showed a higher degree of branching of both the arabinan domain and the mannan core, suggesting some important and unidentified role of Rv1565c in mycobacteria (Driessen *et al.*, 2010).

#### Overview of lipomannan and lipoarabinomannan biosynthesis

##### Synthesis of the mannan core

**Fig. 5:**
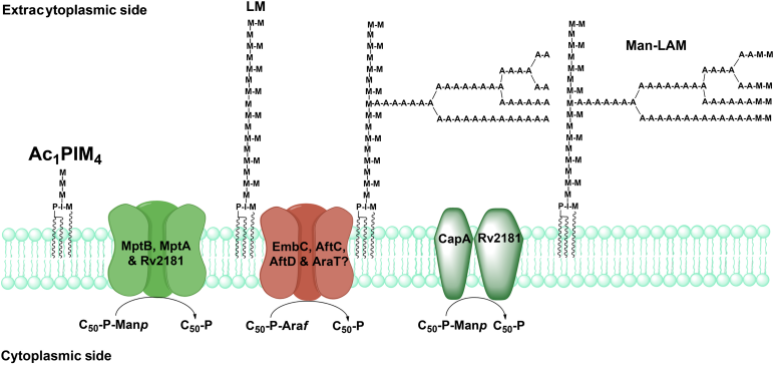
Biogenesis of Man-LAM from *Mycobacterium tuberculosis*. Ac_1_/Ac_2_PIM_4_ plausibly serves as an acceptor and extended by MptB in the α(1→6) direction, followed by MptA and further decorated by singular α(1→2)-Man*p* units by Rv2181 (MptC), resulting in lipomannan. Mature lipomannan is subsequently primed by a singular d-Ara*f* at an unknown position, which is extended by EmbC and/or unidentified α(1→5) arabinofuranosyltransferases. The linear α(1→5)-d-Ara*f* chain is further primed by AftC, which is subsequently extended by AftD and unknown arabinofuranosyltransferases and terminated by the action of AftB to form linear Ara-4 or branched Ara-6. The penultimate Ara*f* of the arabinan domain is further capped by Man*p* residues by CapA and Rv2181 (MptC) to form Man-LAM. For simplicity, only triacylated versions of different lipoglycans are shown.

Using mutant constructs in *C. glutamicum*, and cell-free assays, two α(1→6)-mannopyranosyltransferase activities were reported from *C. glutamicum*, of which one enzyme (*M. tuberculosis* homolog, Rv2174) was characterized as MptA and shown to be involved in the synthesis of the distal end of the α(1→6) mannan backbone of lipomannan (Kaur *et al.*, 2007; Mishra *et al.*, 2007;), while a second α(1→6)-mannopyranosyltransferase, Rv1459c (MptB), was shown to be involved in the synthesis of the proximal end of the mannan backbone and speculated to extend an Ac_1_/Ac_2_PIM_4_ acceptor (Fig. 5) (Mishra *et al.*, 2008a). The deletion of the MptB ortholog in *C. glutamicum* resulted in the absence of lipomannan and lipoarabinomannan and a reduction in α(1→6)-mannopyranosyltransferase activity. Furthermore, cell-free assays involving C_50_-P-Man*p* and heterologously expressed Rv1459c and/or its *M. smegmatis* homolog MSMEG_3120 in *C. glutamicum* showed that these enzymes possessed α(1→6)-mannopyranosyltransferase activity. On this basis, it was suggested that after the transport of Ac_1_PIM_4_ outside the plasma membrane by an unidentified flippase, Mt-MptB catalyzes the addition of further 12–15 Man*p* units (Mishra *et al.*, 2008a). MptB is part of an operon that consists of four ORFs encoding ATP-binding cassette (ABC) transporters (Wang *et al.*, 2006). This enhances a strong possibility for a functional coupling of the glycosyltransferase MptB with ABC transporters, Rv1458c, Rv1457c and Rv1456c. However, a *C. glutamicum* mutant deficient in these ABC transporters showed no difference in their PIM, lipomannan and lipoarabinomannan profiles (L. Eggeling & G.S. Besra, unpublished data), suggestive of gene redundancy, which is not surprising as these ABC transporters are found at multiple locations in the genome of *Corynebacterineae*.

Interestingly, α(1→6) mannan extension is more complex in mycobacteria, based on the evidence that Mt-MptB and Ms-MptB fail to complement the *C. glutamicum*▵*mptB* mutant, suggesting a slightly different substrate specificity of the MptB orthologs of *M. tuberculosis* and *M. smegmatis* as compared with Cg-MptB (Mishra *et al.*, 2008a). Furthermore, the redundancy of Ms-MptB in *M. smegmatis*▵*mptB* indicates that either another as yet unidentified mannopyranosyltransferase is substituting for MptB in the mutant or the distal α(1→6)-mannopyranosyltransferase, MptA, is substituting for the deficiency of Ms-MptB. A mycobacterial strain devoid of MptA and MptB may shed further light on this aspect.

In order to identify the α(1→6)-mannopyranosyltransferase involved in the synthesis of the distal α(1→6) mannan backbone, the homologs of putative glycosyltransferase, Rv2174, were deleted from *C. glutamicum* (Mishra *et al.*, 2007) and *M. smegmatis* (Kaur *et al.*, 2007). The cell wall phenotype of the mutants suggested the accumulation of a truncated lipoglycan (t-LM), deficient in α(1→6)-Man*p* units. A cell-free assay involving C_50_-P-Man*p* and a synthetic disaccharide acceptor, Man-α(1→6)-Man-C_8_, established that the mutant lacked α(1→6)-mannopyranosyltransferase activity and was termed MptA (Mishra *et al.*, 2007). Pfam analysis (Bateman *et al.*, 2004) of the ORF upstream of *mptA* revealed that Rv2173 (putative geranylgeranyl pyrophosphate synthetase, *idsA2*) bears structural similarities to polyprenyl synthetases, which could be functionally related to MptA, and both genes may form a transcriptional unit. Interestingly, mycobacterial MptA contains 13 transmembrane helixes (TMH), of which TMH 3 and 4 are conserved and contain the catalytic DXD motif typified by glycosyltransferases (Liu & Mushegian, 2003), while the C-terminus extracellular loop is nonexistent, unlike other GT-C glycosyltransferases (Zhang *et al.*, 2003; Alderwick *et al.*, 2011;), suggesting the existence of a different model for chain extension as reported already in the case of *M. tuberculosis* EmbC (Shi *et al.*, 2006). Furthermore, the recent genome analysis of *Actinobacterium* and *Micrococcus luteus* identified the presence of homologs of MptA and MptB in a cluster with another gene encoding for a GT-C glycosyltransferase, which are cotranscribed and probably translationally coupled (Young *et al.*, 2010). In contrast, MptA and MptB are dispersed in corynebacteria and mycobacteria.

The α(1→6) mannan core in lipomannan and lipoarabinomannan is further decorated by single α(1→2)-Man*p* branches (Hunter & Brennan, 1990; Chatterjee *et al.*, 1992a;). On the basis of known polyprenol-dependent glycosyltransferases, Rv2181 (MptC), was identified from an 18-kb conserved region and suggested to be involved in the synthesis of α(1→2)-Man*p* side chains of lipomannan (Fig. 5) (Kaur *et al.*, 2006; Kaur *et al.*, 2008; Sena *et al.*, 2010; Mishra *et al.*, 2011;). More recently, it was suggested that MptA and MptC may act in close coordination to synthesize mature lipomannan and lipoarabinomannan, and the length of the mannan core may be regulated by a branching-dependent chain termination mechanism (Sena *et al.*, 2010).

##### Arabinan domain assembly of lipoarabinomannan

Ac_1_/Ac_2_-PIM_2_ is extended by MptB, MptA and MptC to yield a mature lipomannan that probably serves as an acceptor for an uncharacterized arabinofuranosyltransferase to initiate lipoarabinomannan synthesis (Besra *et al.*, 1997). However, the number of arabinofuranosyltransferases required for arabinan domain biosynthesis will depend on the types of arabinan linkage present in mycobacterial lipoarabinomannan (Fig. 5). It is quite likely that mature lipomannan is primed by a few Ara*f* units in a similar fashion as AftA primes the galactan of arabinogalactan in mycobacteria (Alderwick *et al.*, 2006c). However, the enzyme responsible for this activity is not known. The primed Ara*f*-LM is then further extended by EmbC (Rv3793) (Zhang *et al.*, 2003; Shi *et al.*, 2006; Alderwick *et al.*, 2011;) for 12–16 α(1→5)-Ara*f* residues (Birch *et al.*, 2010). Recently, AftC (Rv2673) was shown to introduce the α(1→3)-Ara*f* branch points in both arabinogalactan (Birch *et al.*, 2008) and lipoarabinomannan (Birch *et al.*, 2010). This α(1→3)-Ara*f* branched product, [Ara*f*]_12–16_-LM, is then further extended by an unidentified α(1→5)-arabinofuranosyltransferase.

More recently, Skovierova *et al.* (2009) proposed a second branching α(1→3)-arabinofuranosyltransferase, AftD (Rv0236c). However, unlike the role of AftC as an α(1→3)-arabinofuranosyltransferase, which was experimentally validated by creating a knockout in *M. smegmatis* defective in α(1→3)-arabinofuranosyltransferase activity (Birch *et al.*, 2008), the role of AftD is debatable as Skovierova *et al.* (2009) were unable to create a viable mutant displaying a clear phenotype, and the study was solely based on the usage of artificial chemically defined acceptors using crude *M. smegmatis* and *C. glutamicum* membranes (Skovierova *et al.*, 2009). It is also interesting to note that the same authors also discussed the possibility of AftD as an α(1→5)-arabinofuranosyltransferase involved in α(1→5)-Ara*f* extension of the nonreducing termini of the arabinan domain of lipoarabinomannan and arabinogalactan (Skovierova *et al.*, 2009) (Fig. 5).

The final enzyme involved in arabinan domain biosynthesis is AftB (Rv3805c), which results in a terminal tetra- and hexa-arabinofuranoside structure (Figs 2 and 5). The role of AftB has been experimentally shown to be a β(1→2)-arabinofuranosyltransferase in the synthesis of arabinogalactan (Seidel *et al.*, 2007). However, its role in the synthesis of similar Ara*f* residues in lipoarabinomannan is highly likely after the discovery of a dual role of AftC in arabinogalactan (Birch *et al.*, 2008) and lipoarabinomannan (Birch *et al.*, 2010) biosynthesis.

##### Mannan priming and Man-LAM synthesis

All pathogenic species of the genus *Mycobacterium* are known to possess Man-LAM, which is responsible for some of the immunomodulatory properties of these strains (Briken *et al.*, 2004). A close inspection of the *M. tuberculosis* genome in comparison with *M. smegmatis* that possesses lipoarabinomannan without mannose caps provided the first indication of the role of Rv1635c in Man-LAM biosynthesis. On this basis, the homolog of Rv1635c in *M. tuberculosis* CDC1551 was identified as a glycosyltransferase that could be involved in Man-LAM capping (Dinadayala *et al.*, 2006). Simultaneously, mutants of Rv1635c homologs in *M. marinum* and *M. bovis* BCG showed that the gene encoded for an α(1→5)-mannopyranosyltransferase, CapA, was involved in the addition of the first Man*p* residue on the nonreducing arabinan termini of lipoarabinomannan (Appelmelk *et al.*, 2008). More recently, it was also shown that MptC (Rv2181), which adds α(1→2)-Man*p* residues onto the α(1→6) mannan backbone of lipomannan and lipoarabinomannan, also adds α(1→2)-Man*p* caps at the nonreducing end of lipoarabinomannan in combination with CapA (Kaur *et al.*, 2008) (Fig. 5). Our recent studies with an *M. bovis* BCG mutant defective in *pimE* also suggested its tentative role in α(1→2)-Man*p* capping of Man-LAM (G.S. Besra & B.J. Appelmelk, unpublished data). However, more studies are needed to establish the exact interplay of these mannopyranosyltransferases involved in the mannan caps of Man-LAM.

Almost the entire repertoire of enzymes and genes involved in the biogenesis of lipoarabinomannan and related glycoconjugates has been identified (Table 1), and some of these genes are essential for the survival of *M. tuberculosis*, therefore representing excellent drug targets. However, the roles of lipoarabinomannan and related glycoconjugates in mycobacterial pathogenicity require the availability of mycobacterial mutants defective in their respective biosynthetic pathways, as most of the studies have been carried out using purified molecules that do not represent the true *in vivo* condition during infection. The availability of complete genome sequences of several mycobacteria and related actinomycetes and the development of novel tools for genetic manipulation have enhanced these possibilities.

**Table 1:** Experimentally characterized genes involved in the biosynthesis of LAM and related glycoconjugates

ORF	Function	Role	References
PgsA (Rv2612c)	PI synthase	Synthesis of Phosphatidyl-*myo*-inositol	Jackson *et al.* (2000)
PimA (Rv2610c)	α(1→2)-Mannopyranosyltransferase	Synthesis of PIM_1_	Kordulakova *et al.* (2002)
Rv2611c	Acyltransferase	Synthesis of Ac_1_/Ac_2_-PIM_1_	Kordulakova *et al.* (2003)
PimB' (Rv2188c)	α(1→6)-Mannopyranosyltransferase	Synthesis of Ac_1_/Ac_2_-PIM_2_	Lea-Smith *et al.* (2008); Mishra *et al.* (2008b, 2009)
MgtA/PimB (Rv0557)	α(1→6)-Mannopyranosyltransferase	Synthesis of ManGlcGroAc2 and Ac_1_/Ac_2_-PIM_2_	Tatituri *et al.* (2007a,b); Mishra *et al.* (2008b);
PimC (RvD2-ORF1)	α(1→6)-Mannopyranosyltransferase	Synthesis of Ac_1_/Ac_2_-PIM_3_	Kremer *et al.* (2002)
PimE (Rv1159)	α(1→2)-Mannopyranosyltransferase	Synthesis of Ac_1_/Ac_2_-PIM_5_	Morita *et al.* (2006)
MptB (Rv1459c)	α(1→6)-Mannopyranosyltransferase	Synthesis of proximal mannan backbone i.e. Ac_1_/Ac_2_-PIM_12–17_	Mishra *et al.* (2008a)
MptA (Rv2174)	α(1→6)-Mannopyranosyltransferase	Synthesis of distal mannan backbone i.e. Ac_1_/Ac_2_-PIM_22–25_	Mishra *et al.* (2007)
MptC (Rv2181)	α(1→2)-Mannopyranosyltransferase	Adds α(1→2)-Man*p* units on the mannan backbone, and also adds a second mannose cap on ManLAM	Kaur *et al.* (2008, 2010); Mishra *et al.* (2011)
EmbC (Rv3793)	α(1→5)-Arabinofuranosyltransferase	Involved in the synthesis of the α(1→5)-arabinan backbone	Zhang *et al.* (2003); Alderwick *et al.* (2011);
AftC (Rv2673)	α(1→3)-Arabinofuranosyltransferase	Adds Ara*f* on α(1→5)-arabinan backbone in the α(3→5)-direction	Birch *et al.* (2010)
AftD (Rv0236c)	α(1→3) or α(1→5)-Arabinofuranosyltransferase	Either adds α(1→3)-Ara*f* units to the non-reducing end of the α(1→5)-arabinan branch or synthesize α(1→5) itself	Skovierova *et al.* (2009)
CapA (Rv1635c)	α(1→5)-Mannopyranosyltransferase	Adds first mannose cap on ManLAM	Appelmelk *et al.* (2008)

## Part II – Interactions with host immune system

### Accessibility of lipoglycans to the immune system: localization and trafficking

Lipoarabinomannan and related lipoglycans are not only essential for mycobacterial growth and cell viability (Haites *et al.*, 2005; Kovacevic *et al.*, 2006;), but are also thought to be important in the interactions between the mycobacteria and their host. The nature of these host–pathogen interactions is determined by the accessibility of the lipoglycans to the immune system, i.e. can cell wall-bound lipoglycans be recognized by the pattern-recognition receptors (PRRs) of the immune system and how do the lipoglycans traffic, once released from the mycobacterial cell wall?

The localization of PIMs and lipoarabinomannan in the mycobacterial cell envelope has been assessed in multiple ways, including biotin tagging of lipoarabinomannan and extraction of lipids from the cell wall with detergents or by mechanical treatment with glass beads. Because of the strong conditions needed to extract lipoarabinomannan from the cell wall, and the possibility to detect lipoarabinomannan with lipoarabinomannan-recognizing antibodies on whole cells, it was hypothesized that lipoarabinomannan is firmly attached via its MPI anchor to the surface of the cell (Chatterjee & Khoo, 1998). Biotin labeling, assumed to be restricted to the cell surface, showed two fractions of lipoarabinomannan: one anchored to the cytosolic membrane and one in the mycobacterial outer membrane (mycomembrane) (Hoffmann *et al.*, 2008; Pitarque *et al.*, 2008;). However, biotin is only a small molecule and may have easier access to lipoarabinomannan more buried in the cell wall as compared with the large PRRs, which may reduce the potential of lipoarabinomannan to be recognized by the immune system. Furthermore, native mycobacterial cells are surrounded by a capsule, which could cover lipoarabinomannan. The mycobacterial capsule mainly consists of polysaccharides and proteins (Daffé & Etienne, 1999). Electron microscopy (EM) with immunogold-labeled cells using ConA and anti-arabinan antibodies, combined with nuclear magnetic resonance studies, showed the presence of mannose-capped arabinomannan (Man-AM; i.e. without the lipid anchor present in Man-LAM) in the capsule (Ortalo-Magné*et al.*, 1995). The capsule has been reported to have only a very low lipid content, among which are small amounts of PIMs and virtually no lipoarabinomannan (Ortalo-Magné*et al.*, 1996a,b;). A recent study used immunogold-EM with monoclonal antibodies against PIM_6_ (F183-24), and against the mannose cap (55.92.1A1) and the arabinan domain (F30-5) of Man-AM and Man-LAM to detect surface localization of these lipoglycans. Unperturbed mycobacterial cells bearing an intact capsule display good labeling with these antibodies (Sani *et al.*, 2010), which confirms the presence of PIM_6_ and Man-AM in the capsule. In contrast, mycobacteria without a surrounding capsule due to growth under perturbing conditions (in the presence of 0.05% Tween-80 and mechanical agitation) hardly become labeled with these antibodies. As lipoarabinomannan is localized in the cell wall and not in the capsule, this suggests limited surface exposure of lipoarabinomannan and related glycans buried in the mycomembrane, even if the capsule is not covering the cell wall. However, the amount of lipoarabinomannan or its accessibility in these cells grown under perturbing conditions has not been assessed further.

Although culture filtrate has been reported to contain only trace amounts of lipids (Lemassu & Daffé, 1994), studies with infected macrophages (Mϕ) showed intracellular trafficking of PIMs and lipoarabinomannan, suggesting that these glycolipids are substantially released from mycobacteria. Even release into noninfected bystander cells (Xu *et al.*, 1994; Beatty *et al.*, 2000; Rhoades *et al.*, 2003;) and subsequent presentation through CD1 glycoproteins (Schaible *et al.*, 2000) has been observed. Furthermore, isolated PIMs and lipoarabinomannan can be incorporated into the endomembranes and plasma membranes of different cell types, a process requiring the MPI anchor and the mannan core (Ilangumaran *et al.*, 1995; Shabaana *et al.*, 2005; Welin *et al.*, 2008;). It can be hypothesized that the lipoglycans released are able to modulate the immune response, for example by interfering with phagosome maturation.

### Phagosome maturation arrest

At least two strategies used by *M. tuberculosis* to survive in Mϕ have been described. One is delay of the phagosome maturation, i.e. prevention of fusion of the phagosome with late endosomal and lysosomal organelles, which normally leads to killing and digestion of a pathogen in an acid environment (Armstrong & Hart, 1971; Russell, 2001; Nguyen & Pieters, 2005;). The other strategy is based on escape from the phagosome to the cytosol (van der Wel *et al.*, 2007). In the phagosome maturation arrest, a role for both Man-LAM and PIMs has been implicated (Vergne *et al.*, 2003b; Welin *et al.*, 2008;).

**Fig. 6:**
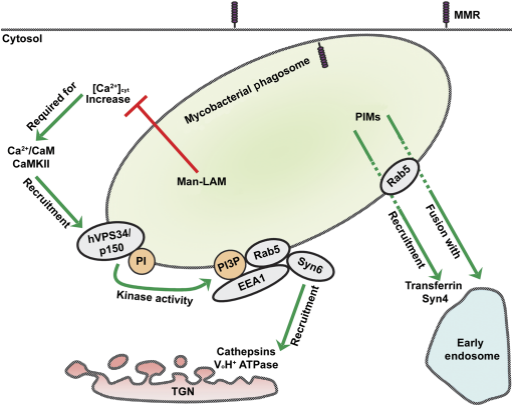
The role of PIMs and Man-LAM in phagosome maturation arrest by mycobacteria. While Man-LAM prevents lysosomal fusion and acidification, PIMs induce fusion with early endosomes to obtain nutrients required for phagosomal residence of mycobacteria. Man-LAM appears to inhibit cytosolic-Ca^2+^ increase and thereby blocks the successive steps of hVPS34 kinase activity at the phagosomal membrane, the recruitment of Rab5, EEA1 and Syn6 to the phagosome, and the delivery of cathepsins and V_o_H^+^ ATPase. The activity of PIMs in phagosome maturation is dependent on Rab5, but the exact mechanism is not known yet. MMR, macrophage mannose receptor; TGN, *trans-*Golgi network; CaM, calmodulin; PI, phosphatidylinositol; PI3P, phosphatidylinositol 3-phosphate; Syn, syntaxin; EEA1, early endosome autoantigen 1.

The phagosome–lysosome fusion process starts after cytosolic Ca^2+^ increase. The Ca^2+^/calmodulin-dependent PI3-kinase hVPS34 and its modulatory subunit p150 will then generate the membrane-trafficking lipid phosphatidylinositol 3-phosphate (PI3P) on the phagosomal membrane (Vergne *et al.*, 2004a). Both PI3P and early endocytic small GTPase Rab5 mediate in the subsequent recruitment of membrane tethering protein early endosome autoantigen 1 (EEA1) to the phagosome (Vergne *et al.*, 2003a) (Fig. 6). EEA1 plays an essential role in phagosome maturation by interacting directly with syntaxin-6, a soluble NSF attachment protein receptor (SNARE) protein involved in the delivery of cathepsins (lysosomal hydrolases) and V_o_H^+^-ATPase from the *trans*-Golgi network to the phagosome (Simonsen *et al.*, 1999). The normal cytosolic Ca^2+^ increase upon an infection is absent during mycobacterial uptake. This has been hypothesized to lead to a reduced activity of the PI3-kinase hVPS34 and an altered production of PI3P in the case of phagocytosis of mycobacteria (Malik *et al.*, 2001; Chua & Deretic, 2004;) (Fig. 6).

This immune evasion strategy of blocking phagosome maturation can be mimicked by Man-LAM, which is also able to inhibit cytosolic Ca^2+^ increase (Fratti *et al.*, 2003b; Vergne *et al.*, 2003b;) (Fig. 6). In Mϕ infected with *M. bovis* BCG or Man-LAM-coated beads, EEA1 is excluded from the early endosome, thereby inhibiting phagosome maturation at the stage of recruitment of late endosomal and lysosomal constituents and, hence, preventing acidification (Fratti *et al.*, 2001). The exact mechanism of [Ca^2+^]_cyt_-modulation by Man-LAM is not known. For *M. tuberculosis* to infect Mϕ without the induction of a cytosolic Ca^2+^ increase, phagocytosis via the complement receptor is required (Malik *et al.*, 2000), but the inhibition of phagosome maturation by Man-LAM appears to involve binding to the MMR instead (Kang *et al.*, 2005). Furthermore, a role for macrophage phosphatase SHP-1 has been suggested, which is activated by Man-LAM and impairs Ca^2+^ signaling (Ono *et al.*, 1997; Knutson *et al.*, 1998; Vergne *et al.*, 2004a;).

Another possibility of interference by Man-LAM in phagosome maturation, distinct from blocking the rise in [Ca^2+^]_cyt_, considers a role for the activation of p38 mitogen-activated protein kinase (p38 MAPK). p38 MAPK activity may indirectly maintain Rab5 in an inactive GDP-bound form (Cavalli *et al.*, 2001; Vergne *et al.*, 2004a;). This is consistent with the report that the induction of p38 MAPK reduces the recruitment of Rab5-effector protein EEA1 to the early endosome (Fratti *et al.*, 2003a). Moreover, the level of p38 MAPK activation is significantly increased upon infection with *M. bovis* BCG (Fratti *et al.*, 2003a) and Man-LAM was hypothesized to be a triggering component (Vergne *et al.*, 2004a). However, recently, it has been shown experimentally that p38 MAPK activation is neither induced nor influenced by isolated Man-LAM, and thus must be linked to other mycobacterial components (Welin *et al.*, 2008).

Noteworthy, lipoarabinomannan is incorporated into membrane lipid rafts, a process that is also required for the phagosome maturation arrest (Welin *et al.*, 2008). Lipid rafts are highly dynamic lipid domains, enriched in cholesterol and glycosphingolipids, and that have been associated with cell signaling (Simons & Toomre, 2000; Pike, 2009;). It has been suggested that the presence of lipoarabinomannan in the endomembrane causes drastic reorganization of the lipid domains and thereby fusion of the lipid vesicles (Hayakawa *et al.*, 2007). However, PI-LAM from avirulent *M. smegmatis* is also incorporated into endomembranes, although to a lesser extent, but it cannot prevent phagosome–lysosome fusion nor inhibit cytosolic Ca^2+^ increase (Vergne *et al.*, 2003b; Kang *et al.*, 2005; Welin *et al.*, 2008;). Given that the MMR only recognizes the mannose-capped Man-LAM and not Ara-LAM or PI-LAM (Schlesinger *et al.*, 1994), this is further evidence that ligation of the Man-LAM to the MMR is required for the phagosome maturation block, which appears to be restricted to the more virulent *Mycobacterium* spp. A role for the MMR has been confirmed recently by showing that glycopeptidolipids from *M. avium* delay phagosome–lysosome fusion by interaction with the MMR and by an MMR siRNA knockdown in human monocyte-derived Mϕ, resulting in increased phagosome–lysosome fusion upon *M. avium* infection (Sweet *et al.*, 2009).

As mammalian phosphoinositide PI3P plays an important role in phagosome maturation, next to lipoarabinomannan, other mycobacterial PI-analogs, PIMs and lipomannan, were investigated as well for their possible interference in phagosome maturation. While for lipomannan no role in phagosome maturation arrest could be detected (Kang *et al.*, 2005), PIMs do have an effect, although in a way distinct from Man-LAM (Vergne *et al.*, 2004b). Similar to lipoarabinomannan, PIMs can be incorporated into lipid rafts and, moreover, the addition of PIMs competitively inhibits lipoarabinomannan insertion (Ilangumaran *et al.*, 1995; Welin *et al.*, 2008;). Although PIMs seem to reverse the effect of lipoarabinomannan of preventing endosomal fusions (Welin *et al.*, 2008), however, indications exist of a different role of PIMs in the phagosome maturation arrest. PIMs do prevent phagosome acidification, but not by reducing the recruitment of syntaxin-6 (Fratti *et al.*, 2003b; Vergne *et al.*, 2004b;). Instead, PIMs induce the acquisition of endosomal SNARE protein syntaxin-4 and the transferrin receptor (Fratti *et al.*, 2003b; Vergne *et al.*, 2004a,b;) (Fig. 6). Transferrin and its receptor are recycling endosomal markers involved in iron delivery (Clemens & Horwitz, 1996; Sturgill-Koszycki *et al.*, 1996;). While Man-LAM arrests phagosome maturation by blocking the recruitment of late endosomal and lysosomal markers, PIMs appear to stimulate fusion with early endosomes and thereby retrieve nutrients necessary for mycobacteria residing in the phagosomal compartments (Kelley & Schorey, 2003; Vergne *et al.*, 2004b;). This process is also Rab5-dependent (Gorvel *et al.*, 1991), in particular when Rab5 activity is rate limiting, but whether PIMs affects Rab5 directly or indirectly is not yet known (Vergne *et al.*, 2004b).

Interestingly, also in the effect that PIMs exert on the phagosome maturation, the MMR seems to play a role. While the lower-order PIMs (PIM_2_) are not recognized by the MMR, the MMR has a high affinity for higher-order PIMs (PIM_5_ and PIM_6_) (Torrelles *et al.*, 2006). This is consistent with the report that only in Mϕ stimulated with higher-order PIMs was a significant increase in phagosome-lysosome fusion seen upon MMR blockade (Torrelles *et al.*, 2006). Thus, although PIMs and Man-LAM influence the phagosome maturation by distinct mechanisms, both may involve recognition by the MMR. This indicates a balance between Man-LAM preventing maturation into the phagolysosome on the one hand and PIMs stimulating early endosomal fusion to retrieve nutrients on the other (Vergne *et al.*, 2004b).

Inhibition of phagosome maturation by pathogenic *Mycobacterium* spp. may be a critical first step for their intracellular survival. Mycobacteria probably display several mechanisms to prevent lysosomal transfer, of which one is interference of Man-LAM in the phagosome maturation process. A *M. marinum* mutant that only produces lipoarabinomannan devoid of mannose caps showed a significant increase in colocalization with phagolysosomes in murine Mϕ as compared with its parent strain. However, the absolute numbers remained low in this assay (7.4%, 12.0% and 5.0% for the wild-type, mutant and complemented strain, respectively), and importantly, no significant differences in bacterial survival were observed (Appelmelk *et al.*, 2008). Other mechanisms of phagosome maturation blockade, independent of Man-LAM, have been reported, for example the secretion of SapM by *M. tuberculosis*, a lipid phosphatase that hydrolyzes the PI3P on the endomembranes (Vergne *et al.*, 2005), and the secretion of a eukaryotic-like serine/threonine protein kinase G (PknG) (Walburger *et al.*, 2004).

### Clusters of differentiation (CD)1

CD-1 glycoproteins have been identified as important antigen-presenting molecules of the immune system, next to major histocompatibility complex (MHC) class I and II molecules. While MHC class I and II present peptide antigens, CD1 molecules present glycolipids, thereby covering the presentation of a large variety of both self as well as microbial antigens (Young & Moody, 2006; de Libero & Mori, 2009;). In mycobacterial infection, CD1 ensures the presentation of the glycolipids unique to the mycobacterial cell wall to activate CD1-restricted T cells and is thereby involved in shaping the immune response (Porcelli *et al.*, 1998; Sieling *et al.*, 1999; Barral & Brenner, 2007;).

Human CD1 molecules are expressed by a variety of antigen-presenting cells (APC) and can be divided into three groups: CD1a, CD1b and CD1c together form group 1, and CD1d and CD1e form group 2 and group 3, respectively. Murine homologs for group 1 CD1 molecules have not been identified, but mice do express CD1d. Group 1 CD1 molecules present lipids to a clonally diverse T-cell population in which the precursors have unique specificity for a single antigen (Barral & Brenner, 2007). The expression of group 1 CD1 on isolated human myeloid APC is hardly detectable, but it is upregulated to high levels within a couple of days after infection with *M. tuberculosis* or activation by mycobacterial lipids (Roura-Mir *et al.*, 2005) (Felio *et al.*, 2009). This demonstrates an apparent role of antigen presentation by the group 1 CD1 in the clonal expansion of T cells and, hence, the adaptive immune response against mycobacterial infection (Roura-Mir *et al.*, 2005; Barral & Brenner, 2007;). CD1d presents lipids to CD1d-restricted natural killers T (NKT) cells including the subset of clonally less diverse invariant NKT cells that display a rapid innate-like response (Barral & Brenner, 2007). In contrast to group 1 CD1, CD1d molecules are constitutively expressed and are reported to be downregulated during mycobacterial infection, confirming their association with the innate immune response (Roura-Mir *et al.*, 2005; Moody, 2006;). CD1e is only restricted to myeloid dendritic cells (DCs) and is not expressed at the cell surface and thus does not present antigens to TCRs. In this review, we focus on CD1b and CD1d, because these CD1 molecules bind and present PIMs and related lipoglycans. Group 2 CD1d has been reported to only bind lower-order PIMs, PIM_2_ and PIM_4_, but not lipomannan or Man-LAM (Fischer *et al.*, 2004; Zajonc *et al.*, 2006;). In contrast, group 1 CD1b binds several mycobacterial lipid including PIM_2_ and Man-LAM (Sieling *et al.*, 1995; Prigozy *et al.*, 1997; Ernst *et al.*, 1998;).

The structure of CD1 molecules has similarities to the MHC class I molecules, but shows some important differences in its binding groove, which is deeper and facilitates the binding of two acyl chains as present in the MPI anchor of PIMs and Man-LAM (Zeng *et al.*, 1997; Porcelli *et al.*, 1998; Fischer *et al.*, 2004;). While the lipid anchoring in the hydrophobic CD1 groove is relatively nonspecific, the TCR recognizes the hydrophilic carbohydrate head group of the antigens with high specificity (Moody *et al.*, 1997). As compared with CD1b, additional interactions between the center of the binding groove of CD1d and the polar head group of the PIM_2_ are of additive importance for the formation of a stable glycolipid complex and subsequent T cell recognition (Zajonc *et al.*, 2006). In the presentation of PIM_4_ by CD1d, the two additional α(1→6)-linked Man*p* residues are probably orientated away from the binding groove (Zajonc *et al.*, 2006). Considering the low abundance of PIM_4_ in the mycobacterial cell wall in contrast to (diacylated) PIM_2_ (Gilleron *et al.*, 2001), presentation of PIM_4_ by CD1d may not be of high biological significance. For group 1 CD1b, mycobacterial antigens with head groups much larger than present in PIM_2_ have been described, which raises questions regarding how these large carbohydrates fit in the narrow space between the TCR and CD1 (Young & Moody, 2006). Higher-order PIM_6_ needs processing into the smaller PIM_2_ before being able to stimulate CD1b-restricted T cells. A role in this antigen processing has been implicated for CD1e, because the presence of CD1e is required for the activation of CD1b-restricted T cells by PIM_6_, and not by PIM_2_ (de la Salle *et al.*, 2005). As mentioned above, CD1e does not present lipid antigens at the cell surface, but probably aids in endosomal/lysosomal α-mannosidase activity to produce PIM_2_ by binding PIM_6_ similar to the other antigen-presenting CD1 molecules (de la Salle *et al.*, 2005). Secondly, CD1e may facilitate the loading of other CD1 molecules (de Libero & Mori, 2009). How Man-LAM is presented in the interaction between the TCR and the CD1b-Man-LAM complex has not yet been resolved. Man-LAM may be partly digested similar to PIM_6_ (Ernst *et al.*, 1998), which is most likely, as already PIM_6_ with its short carbohydrate head group requires processing. Of note, CD1b and Man-LAM do colocalize in the cell (Prigozy *et al.*, 1997) and CD1b is able to bind Man-LAM (Ernst *et al.*, 1998). Two other options have been suggested by Young & Moody (2006). One possibility is flattening of the glycan part of Man-LAM between the TCR and CD1, so that only one or two carbohydrate units are positioned directly between the TCR and CD1. Multiple TCR-docking orientations may play a role in this as well. In the second option, Man-LAM is not presented by CD1b, but stimulates the process of CD1-dependent T cell activation indirectly via the mechanisms discussed below (Young & Moody, 2006).

CD1b is the predominant group 1 CD1 molecule present in the late endosomes/lysosomes and MHC class II compartments (Prigozy *et al.*, 1997; Ernst *et al.*, 1998; Schaible *et al.*, 2000;) and shares with MHC class II molecules the requirement for acidification in order to function (Benaroch *et al.*, 1995; Sugita *et al.*, 1999;). PIMs and Man-LAM have been observed to be released in the phagosomes of infected cells and transported into the same intracellular compartments (Xu *et al.*, 1994; Prigozy *et al.*, 1997; Schaible *et al.*, 2000;). The low pH in these compartments causes conformational changes in the structure of CD1b including relaxation of certain parts of its binding groove to facilitate subsequent antigen loading (Ernst *et al.*, 1998; Sugita *et al.*, 1999; Kronenberg & Sullivan, 2008; Relloso *et al.*, 2008; de Libero & Mori, 2009;). As described above, PIMs and Man-LAM interfere in phagosome maturation and in particular Man-LAM has been shown to prevent endosomal acidification (Fratti *et al.*, 2001). Hence, PIMs and Man-LAM likely impede their own presentation by CD1b. On the other hand, mycobacterial lipids have been shown to induce the transcription and expression of group 1 CD1 glycoproteins at the surface of the APC by signaling through TLR-2 (Roura-Mir *et al.*, 2005; Moody, 2006;). Possible lipids involved were reported to be PIM_2_ and Ara-LAM extracted from the mycobacterial cell wall (Roura-Mir *et al.*, 2005). However, PIM_2_ and Ara-LAM are poor TLR2 ligands as discussed in the next section (Nigou *et al.*, 2008). Copurified lipopeptides, which are more potent inducers of TLR2 signaling, may also have induced CD1 expression in this assay (Nigou *et al.*, 2008; Zahringer *et al.*, 2008;).

Mycobacteria are able to interfere with the immune response against mycobacterial infection including the modulation of peptide antigen presentation by MHC class I and II molecules (Kaufmann & Schaible, 2005). Therefore, the lipid antigen presentation via four different CD1 glycoproteins forms an important alternative mechanism to induce an effective immune response (Sugita *et al.*, 1999). Although the function and expression of CD1 molecules can be impaired by mycobacteria or mycobacterial components such as capsular α-glucan (Gagliardi *et al.*, 2007, 2009; Balboa *et al.*, 2010), the many distinct pathways for antigen sampling from various intracellular localizations and their subsequent presentation circumvents the immune evasion strategies exploited by mycobacteria (Sugita *et al.*, 1999; Kaufmann & Schaible, 2005; de Libero & Mori, 2009;). Furthermore, both group 1 and group 2 CD1 presentation of lipid antigensseem to play a potential role in the protection against tuberculosis by vaccination with BCG (Watanabe *et al.*, 2006; Venkataswamy *et al.*, 2009;).

### TLRs

Three TLRs have been implicated to play a role in the mycobacterial infection: TLR2, TLR4 and TLR9 (Ozinsky *et al.*, 2000; Quesniaux *et al.*, 2004a; Jo, 2008;). PIMs, lipomannan and lipoarabinomannan have all been examined for signaling via TLR2 and via TLR4, of which an overview is given here.

Lipoproteins are the major ligands for TLR2 (Brightbill *et al.*, 1999), but MPI-anchored mannosylated lipoglycans can signal via TLR2 as well, depending on their degree of acylation and mannosylation (Gilleron *et al.*, 2006; Doz *et al.*, 2007; Nigou *et al.*, 2008;). TLR2 dimerizes with either TLR1 or TLR6 in order to function (Ozinsky *et al.*, 2000). TLR1/TLR2 heterodimers mainly recognize triacylated lipoproteins, while the diacylated forms bind TLR2/TLR6 (Takeuchi *et al.*, 2002; Akira & Takeda, 2004;). Lipoglycan-induced signaling occurs via the TLR1/TLR2 complex (Elass *et al.*, 2005; Gilleron *et al.*, 2006; Nigou *et al.*, 2008;). A positive relation exists between the length of the mannan chain and the ability of the lipoglycan to activate TLR2 (Nigou *et al.*, 2008). The lipoglycan bearing the largest accessible mannan chain (i.e. not substituted with an arabinan domain) – lipomannan – showed to be a potent inducer of TLR2-signaling (Quesniaux *et al.*, 2004b), although this activity is restricted to the tri- and tetra-acylated forms (Ac_1_/Ac_2_LM) (Gilleron *et al.*, 2006; Doz *et al.*, 2007;). Next to the induction of cytokines, Mϕ stimulated with lipomannan displayed increased production of matrix metalloproteinase (MMP)-9 (Elass *et al.*, 2005). This was due to the downregulation of the transcription of the MMP-9 inhibitor, tissue inhibitor of metalloproteinases-1, and dependent on TLR2. This implies a role for lipomannan in tissue destruction by MMP-9 during mycobacterial infection via interaction with TLR2 (Elass *et al.*, 2005). Furthermore, lipomannan induces granuloma macrophage fusion in an *in vitro* granuloma model in a TLR2-dependent way (Puissegur *et al.*, 2007).

In the group of PIMs, both Ac_1_/Ac_2_PIM_2_ and Ac_1_/Ac_2_PIM_6_ have been reported to signal via TLR2, irrespective of their acylation pattern (Jones *et al.*, 2001; Gilleron *et al.*, 2003;). Further, in two studies, cellular activation via TLR2 by non-mannose-capped lipoarabinomannan (PI-LAM/Ara-LAM) from rapidly growing species has been observed (Means *et al.*, 1999; Underhill *et al.*, 1999;), but not for *M. tuberculosis* or BCG-derived Man-LAM. In addition, an inflammatory response induced by PI-LAM from *M. smegmatis* in mice appeared to be TLR2 dependent (Wieland *et al.*, 2004). However, in a comparative study of all lipoglycans, Ac_1_/Ac_2_PIM_2_, PI-LAM and Ara-LAM were shown to be poor inducers of TLR2 signaling as compared with lipomannan and Ac_1_/Ac_2_PIM_6_ (Nigou *et al.*, 2008). This is consistent with an earlier study showing that in contrast to lipomannan, neither Ara-LAM from *M. chelonae* nor Man-LAM and Ac_1_/Ac_2_PIM_2_ from *Mycobacterium kansasii* mediate TLR2-dependent activation (Vignal *et al.*, 2003). Moreover, chemical degradation of the arabinan domain of Man-LAM from *M. kansasii* restored its ability to induce cytokine secretion via TLR2, which suggests that the arabinan domain prevents the proper interaction of Man-LAM with TLR2 (Vignal *et al.*, 2003). This was confirmed by a recent study by Birch *et al.* (2010) in which lipoarabinomannan containing a truncated arabinan domain from an *M. smegmatis* AftC knockout mutant showed enhanced TLR2 signaling as compared with wild-type lipoarabinomannan. The positive effects of lipoarabinomannan in the earlier reports could be due to contamination of the lipoarabinomannan extract with lipopeptides (Nigou *et al.*, 2008; Zahringer *et al.*, 2008; Geurtsen *et al.*, 2009; Birch *et al.*, 2010;). Overall, the data indicate that lipomannan, and in a minor respect PIM_6_, are the only significant TLR2 ligands from this group of mycobacterial lipoglycans.

For TLR4, only a few mycobacterial lipoglycans have been reported as ligands. Using nonactivated Mϕ, only tri- and tetra-acylated lipomannan from *M. tuberculosis* and *M. bovis* BCG, respectively, show signaling via TLR4 (Gilleron *et al.*, 2006; Doz *et al.*, 2007;). In contrast to TLR2, which can only signal by the recruitment of adaptor proteins, Myeloid differentiation primary response gene (MyD)-88 and Toll-interleukin 1 receptor (TIR) domain-containing adapter protein (TIRAP), TLR4 can also signal via TIR-domain-containing adapter-inducing interferon-β and translocating chain-associating membrane protein (Akira & Takeda, 2004; Jo, 2008;). The secretion of proinflammatory cytokines, by Mϕ stimulated with lipomannan, is strongly dependent on the MyD88/TIRAP pathway (Doz *et al.*, 2007). Most probably, cell wall-associated or soluble factors other than lipoglycans [e.g. heat shock proteins (Bulut *et al.*, 2005)] stimulate TLR4 signaling in *M. tuberculosis* infection; while both live and ‘heat-killed’*M. tuberculosis* activate cells via TLR2, only live *M. tuberculosis* induces TLR4-dependent activation excluding a major role for the heat-stable glycolipids (Means *et al.*, 1999). In Table 2, an overview is given of all TLR signaling observed for lipomannan using TLR1/2/4/6 knockout cells or antibodies against specific TLRs.

**Table 2:** Overview of reports on the activation of TLR2 by LM

LM type	Cells	Dependence shown for	Effect	References
Mc-LM, Mk-LM	THP-1 Mϕ	TLR2+CD14	Release TNF and IL-8	Vignal *et al.* (2003)
Mc-LM, Mk-LM, BCG-LM	BMDMϕ	TLR2	Release TNF and NO	Quesniaux *et al.* (2004b)
Mc-LM, Mk-LM, BCG-LM	THP-1 Mϕ	TLR2	expression MMP-9	Elass *et al.* (2005)
BCG-LM	THP-1, BMDMϕ	TLR1/2	Release TNF	Gilleron *et al.* (2006)
BCG-Ac_1_LM	THP-1, BMDMϕ	TLR1/2, TLR4	Release TNF	
Mc-LM, Mk-LM, Ms-LM, Mtb-LM	human PBMC	TLR2	Mϕ fusion into MGC in *in vitro* granuloma	Puissegur *et al.* (2007)
BCG-Ac_1_LM	BMDMϕ	TLR2, TLR4	Release TNF and NO	Doz *et al.* (2007)
BCG-Ac_2_LM	BMDMϕ	TLR4	Release TNF and NO	
Mtb-Ac_1(2)_LM	BMDMϕ	TLR4	Release TNF	
Mtb-LM, BCG-LM	HEK/CD14/TLR2, THP-1 Mϕ	TLR1/2+CD14	Activation NF-κB	Nigou *et al.* (2008)

Ms, *Mycobacterium smegmatis*; Mk, *Mycobacterium kansasii*; Mc, *Mycobacterium chelonae*; Mtb, *Mycobacterium tuberculosis*; BCG, *Mycobacterium bovis* BCG; Ac_1_/Ac_2_LM, tri-/tetra-acylated LM (*note*: in some articles written as Ac_3_/Ac_4_LM); BMDMϕ, bone marrow-derived macrophages; PBMC, peripheral blood mononuclear cells; MGC, multinucleated giant cells.

A few studies report on an immunosuppressive effect of lipomannan, Ac_1_LM and PIMs on lipopolysaccharide-activated Mϕ (Quesniaux *et al.*, 2004b; Doz *et al.*, 2007, 2009). These inhibitory effects were independent of their signaling via TLR2, which partially compensated the decrease of proinflammatory cytokine secretion (Quesniaux *et al.*, 2004b; Doz *et al.*, 2007;). Which receptor-lipoglycan ligation is then responsible for the immunosupression is not yet known, but it is unlikely to be the MMR (Doz *et al.*, 2007): in contrast to the higher-order PIMs, the MMR recognizes lipomannan and Ac_1_/Ac_2_PIM_2_ only with low affinity (Torrelles *et al.*, 2006), while they show similar inhibitory effects on lipopolysaccharide-activated Mϕ (Doz *et al.*, 2009). Importantly, neither *lyso*-PIM (monoacylated PIM) nor PI were inhibitory in this assay, indicating the requirement of mannosyl moiety and a diacylated MPI anchor (Doz *et al.*, 2009). Of note, in all these experiments, lipopolysaccharide was used, which is a major ligand for TLR4. PIMs might interfere in the interaction between lipopolysaccharide and TLR4, thereby causing its inhibitory effect, but this seems unlikely (Doz *et al.*, 2009). However, lipopolysaccharide is a nonmycobacterial ligand, which makes this assay system rather artificial for the comprehension of TLR-dependent immunomodulation by PIMs and related lipoglycans during mycobacterial infection. Finally, the immunomodulatory properties of Man-LAM on both lipopolysaccharide-activated Mϕ and DCs have been described as well (Geijtenbeek *et al.*, 2003; Pathak *et al.*, 2005;). These effects of Man-LAM can be attributed to its binding by the MMR and DC-SIGN, respectively, which will be discussed in the next sections.

*In vivo* studies using TLR knockout mice are not fully in agreement on whether TLRs are crucial for mycobacterial clearance and host defense (Drennan *et al.*, 2004; Ferwerda *et al.*, 2005;) or not (Sugawara *et al.*, 2003a,b; Nicolle *et al.*, 2004; Holscher *et al.*, 2008;). Importantly, TLR signaling via the MyD88 pathway has been shown in multiple studies to be dispensable for the induction of an efficient adaptive immune response (Fremond *et al.*, 2004; Nicolle *et al.*, 2004; Ryffel *et al.*, 2005; Holscher *et al.*, 2008;). A critical role for TLRs then has been hypothesized in the early recognition and killing of mycobacteria (Quesniaux *et al.*, 2004a; Ryffel *et al.*, 2005; Jo, 2008;). However, this innate immune response mainly depends on MyD88 and may also involve a MyD88-mediated pathway different from via TLR (Feng *et al.*, 2003; Holscher *et al.*, 2008;), which is the type I interleukin-1 receptor (IL-1R1)-mediated signaling (Fremond *et al.*, 2007; Reiling *et al.*, 2008;). Alternatively, TLR2 activation may have a second function as a regulator by preventing an exaggerated inflammatory immune response in a later stage of mycobacterial infection (Drennan *et al.*, 2004; Sutmuller *et al.*, 2006; Jo, 2008; Harding & Boom, 2010;). Pattern recognition by multiple TLRs does contribute to the control of mycobacterial infection (Bafica *et al.*, 2005; Ryffel *et al.*, 2005; Jo *et al.*, 2007;), but may play a less significant role than originally assigned (Reiling *et al.*, 2008).

### DC-SIGN

In contrast to the interaction between mycobacteria and Mϕ, which can be mediated by several different receptors (e.g. complement receptors, CD14, MMR, scavenger receptor) (Ernst, 1998), binding of mycobacteria to DCs is for the largest part mediated by DC-SIGN (CD209) (Tailleux *et al.*, 2003). DC-SIGN is a type II transmembrane tetrameric C-type lectin containing one carbohydrate recognition domain per monomer. It was originally hypothesized that via interaction with DC-SIGN, mycobacteria modulate the immune response and thereby escape immune surveillance (Geijtenbeek *et al.*, 2003; van Kooyk & Geijtenbeek, 2003;). However, the exact role of DC-SIGN in tuberculosis infection has not yet been clarified and is still under debate (Neyrolles *et al.*, 2006; Ehlers, 2009; Tanne & Neyrolles, 2010;). Studies on the relation between the level of expression of DC-SIGN in human populations and susceptibility to tuberculosis are contradicting each other, varying from a protective effect to increased susceptibility to tuberculosis by high DC-SIGN expression or no correlation at all (Barreiro *et al.*, 2006; Gomez *et al.*, 2006; Ben-Ali *et al.*, 2007; Vannberg *et al.*, 2008;).

In a binding study using a DC-SIGN-expressing cell line, the lectin showed a high preference for the *Mycobacterium* spp. from the tuberculosis complex, while it bound little or not to strains from outside the complex (Pitarque *et al.*, 2005). On the basis of differences in the mannose-capping degree of lipoarabinomannan between these species and the fact that DC-SIGN only recognizes lipoarabinomannan if it is mannose-capped, it was expected that Man-LAM would establish the interaction between mycobacteria and DC-SIGN as present on DCs (Geijtenbeek *et al.*, 2003; Maeda *et al.*, 2003;). Furthermore, DC-SIGN has a high-affinity α(1→2)-linked Man*p* residue, which increases with chain length (Koppel *et al.*, 2004). The predominant mannose cap on Man-LAM of *M. tuberculosis* and *M. bovis* BCG consists of α(1→2)-linked dimannosides, and α(1→2)-linked trimannosides are also present (Pitarque *et al.*, 2005). Surprisingly, an *M. bovis* BCG mutant, which only produces lipoarabinomannan without mannose cap, did not bind less to DC-SIGN and DCs as compared with wild-type BCG (Appelmelk *et al.*, 2008); thus, ligands for DC-SIGN other than Man-LAM must be present in the cell wall.

Next to Man-LAM, PIMs have been examined for their binding to DC-SIGN as well (Torrelles *et al.*, 2006; Driessen *et al.*, 2009;). In particular, the higher-order PIMs (PIM_5_ and PIM_6_), with terminal α(1→2)-linked Man*p* residues reminiscent of the mannose cap of Man-LAM, were of interest. As expected, DC-SIGN recognizes the higher-order PIMs with high affinity as compared with the lower-order PIMs (Boonyarattanakalin *et al.*, 2008; Driessen *et al.*, 2009;). Besides PIMs, other potential ligands in the mycobacterial cell wall for DC-SIGN have been identified: lipomannan, Man-AM and mannosylated lipoproteins 19 and 45 kDa (Pitarque *et al.*, 2005), and more recently, capsular α-glucan (Geurtsen *et al.*, 2009) (a list is provided in Table 3). All these compounds have been shown to bind DC-SIGN and/or inhibit binding of *M. tuberculosis* to DC-SIGN. This suggests that binding of mycobacteria to DC-SIGN and DCs is not mediated by one dominant ligand, but that there is a redundancy in ligands for DC-SIGN present on the cell surface (Pitarque *et al.*, 2005). This was supported by the observation that even a double knockout *M. bovis* BCG strain that neither produces the mannose cap on lipoarabinomannan nor higher-order PIMs did not show any reduction in binding to DC-SIGN and DCs as compared with its parent strain (Driessen *et al.*, 2009). Moreover, this mutant strain did not induce an altered cytokine profile in DCs (Driessen *et al.*, 2009). All of the reported ligands for DC-SIGN can be found in DC-SIGN nonbinding *Mycobacterium* spp. as well. Hence, other factors probably determine the binding to DC-SIGN instead of only the presence or the absence of one or a few ligands. The affinity of DC-SIGN for specific *Mycobacterium* spp. may be related to differences in the mannosylation pattern of potential ligands, the relative abundance of various compounds at the cell surface (in particular, α(1→2)-linked Man*p-*containing compounds), cell wall structure and/or capsule composition. Finally, not all potential ligands, when cell wall bound, may be accessible to DC-SIGN and thus of equal importance for the binding of mycobacteria to DC-SIGN.

**Table 3:** Overview of reported mycobacterial ligands for DC-SIGN

Mycobacterial ligands for DC-SIGN	Discussed in
ManLAM	Geijtenbeek *et al.* (2003); Pitarque *et al.* (2005); Appelmelk *et al.* (2008)
ManAM	Pitarque *et al.* (2005)
LM	Pitarque *et al.* (2005)
(higher-order) PIMs	Torrelles *et al.* (2006); Boonyarattanakalin *et al.* (2008); Driessen *et al.* (2009)
19- and 45-kDa antigen*	Pitarque *et al.* (2005)
Mannosylated proteins†	Driessen *et al.* (2009)
Capsular α-glucan	Geurtsen *et al.* (2009)

* Mannosylated lipoprotein (19 kDa) and mannosylated glycoprotein (45 kDa).

† Unidentified proteins from *Mycobacterium bovis* BCG whole-cell lysates probed SDS-PAGE/immunoblot with a DC-SIGN-Fc construct. *Note*: a ConA affinity capture assay identified >30 putative mannosylated proteins in *Mycobacterium tuberculosis* culture filtrate (Gonzalez-Zamorano *et al.*, 2009), which constitute potential ligands for DC-SIGN.

To investigate the DC-SIGN-dependent modulation of the immune response by mycobacteria or their mannosylated lipoglycans, several *in vitro* and *in vivo* studies have been performed. Purified mycobacterial Man-LAM, not Ara-LAM, inhibits both lipopolysaccharide- as well as BCG-induced maturation of DCs by reducing the expression of MHC class II and costimulatory molecules (CD80, CD83 and CD86) as compared with untreated lipopolysaccharide- or BCG-activated cells (Geijtenbeek *et al.*, 2003). Furthermore, Man-LAM has been shown to induce the secretion of anti-inflammatory IL-10 in lipopolysaccharide-activated DCs (Geijtenbeek *et al.*, 2003). Ligation of Man-LAM to DC-SIGN appears to interfere with lipopolysaccharide signaling via TLR4 by the activation of serine-threonine kinase Raf-1, which subsequently leads to acetylation of NF-κB subunit p65. Acylation of p65 prolongs and enhances the transcription of *IL10*, thereby increasing IL-10 production (Gringhuis *et al.*, 2007). Together with a study on mice lacking SIGN receptor (SIGNR)-1, a murine homolog for DC-SIGN, which displayed a stronger T helper 1 response upon *M. tuberculosis* infection and a reduced level of IL-10 production (Wieland *et al.*, 2007), this led to the initial thought that the interaction with DC-SIGN is beneficial for mycobacteria.

Recently, it has been shown that Man-LAM alters the cytokine profile in lipopolysaccharide-activated DCs by increasing the secretion of not only IL-10, but also of proinflammatory IL-12 and IL-6 (Gringhuis *et al.*, 2009). Binding of Man-LAM or mannose-containing pathogens like *M. tuberculosis* induces the recruitment of effector proteins to a DC-SIGN signalosome, which is required for the activation of Raf-1, followed by enhancement of the transcription of the genes encoding IL-12 and IL-6 in a similar manner as for IL-10 (Gringhuis *et al.*, 2009). Noteworthy, lipopolysaccharide is a nonmycobacterial ligand and both Man-LAM (Geijtenbeek *et al.*, 2003) as well as intact mycobacteria (Driessen *et al.*, 2009) do not induce IL-10 secretion in nonactivated DCs. In the context of mycobacterial infection, further research on interference of DC-SIGN signaling induced by Man-LAM with signaling via TLR2 by for example BCG-Ac_1_LM or the 19 kDa antigen would be of interest.

A study using mouse DCs showed a higher induction of suppressor of cytokine signaling-1 expression by SIGNR1 stimulation as compared with signaling via TLR2 (Srivastava *et al.*, 2009). It is important to mention that seven murine homologs for human DC-SIGN are known. Each homolog differs in certain properties from the human DC-SIGN, among which are the specific mannose- and fucose-structures it recognizes, and this may alter their functions significantly (Powlesland *et al.*, 2006; Tanne *et al.*, 2009;). Strikingly, human DC-SIGN transgenic mice exhibited decreased pathology and prolonged survival during mycobacterial infection (Schaefer *et al.*, 2008). A clear protective role has recently also been shown for the murine ortholog SIGNR3 (Tanne *et al.*, 2009). Resistance to *M. tuberculosis* infection was impaired in SIGNR3-deficient mice, but not in mice lacking SIGNR1 and SIGNR5 (Tanne *et al.*, 2009).

**Fig. 7:**
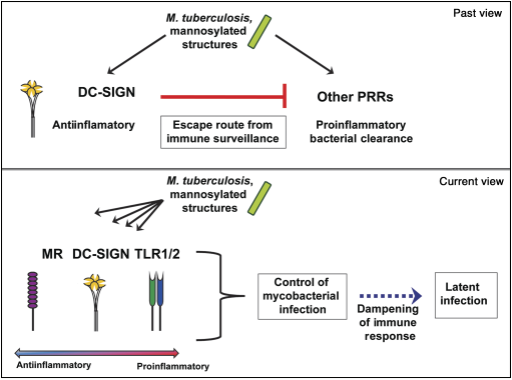
The role of DC-SIGN and other PRRs in the immune response against mycobacterial infection. Previously, it was hypothesized that binding to C-type lectin DC-SIGN forms an escape route for mycobacteria to immune surveillance by interfering with signaling via other PRRs. Currently, signaling via PRRs such as DC-SIGN, MMR and TLR2 has been suggested to have a dual function, with both a role in bacterial clearance or induction of proinflammatory cytokines, as well as in preventing an exaggerated immune response. While their main function is then in the protection of the host, *Mycobacterium tuberculosis* benefits from binding to or signaling via these PRRs as well, which may promote latent mycobacterial infection instead of complete bacterial clearance.

DC-SIGN specifically recognizes the pathogenic *Mycobacterium* spp. from the tuberculosis complex (Pitarque *et al.*, 2005). By expressing many ligands for DC-SIGN, DC-SIGN is the most important uptake receptor for mycobacteria to infect DCs. However, concluding from these last studies, DC-SIGN-binding appeared not to be mainly beneficial for the pathogens, and DC-SIGN may function primarily in the protection of the host. How DC-SIGN signaling exactly optimizes the immune response against mycobacteria, i.e. by strengthening the proinflammatory response or by preventing excessive inflammation, remains to be established (Ehlers, 2009) (Fig. 7).

### Mannose receptor

The MMR (CD206) is a type I transmembrane monomeric C-type lectin with eight carbohydrate recognition domains. In addition to the complement receptors, the MMR mediates for a large part in the phagocytic uptake of mycobacteria by Mϕ (Schlesinger, 1993). The MMR recognizes virulent *M. tuberculosis* strains Erdman and H37Rv, but not avirulent H37Ra, which has been suggested to be caused by subtle structural differences in Man-LAM (Schlesinger, 1993; Schlesinger *et al.*, 1996;). Furthermore, the MMR also shows a low affinity for nontuberculosis mycobacteria *M. smegmatis*, *M. phlei* and *M. kansassi*, of which the first two species only bear lipoarabinomannan without a mannose cap (Astarie-Dequeker *et al.*, 1999). Although the presence of a mannose cap on Man-LAM is essential for the recognition of lipoarabinomannan by the MMR (Schlesinger *et al.*, 1994), it does not completely explain the differences in MMR-mediated uptake by Mϕ between the *Mycobacterium* spp. (Schlesinger *et al.*, 1996; Astarie-Dequeker *et al.*, 1999; Appelmelk *et al.*, 2008;). Next to the recognition of the mannose cap on Man-LAM, the MMR furthers binds higher-order PIM_5_ and PIM_6_ with a preference of the triacylated species above the tetra-acylated ones, and glycopeptidolipids, but not to lower-order PIM_2_, lipomannan and Ara-LAM or PI-LAM (Schlesinger *et al.*, 1994; Villeneuve *et al.*, 2005; Torrelles *et al.*, 2006;). Because of their structural similarities, mannosylated proteins and (phosphorylated) arabinomannans and mannan may be involved as well (Ortalo-Magné*et al.*, 1995; Dobos *et al.*, 1996; Maes *et al.*, 2007; Torrelles *et al.*, 2008a;). Man-LAM and related lipoglycans all contribute to the MMR-mediated phagocytosis of mycobacteria (Villeneuve *et al.*, 2005; Torrelles *et al.*, 2008b;). Overexpression of Mt-ManB in *M. smegmatis* results in the overproduction of PIMs, lipomannan and lipoarabinomannan. Subsequent increased association with Mϕ is probably due to the higher amount of higher-order PIMs, as lipoarabinomannan from *M. smegmatis* does not have a mannose cap (McCarthy *et al.*, 2005).

A role for the MMR has been implicated in many of the immunomodulatory effects by Man-LAM and the related glycans described above. Next to uptake of mycobacteria by Mϕ via the MMR, the MMR is also involved in the transport of glycolipids to uninfected bystanders cells and intracellular trafficking to the late endosomal compartments (Prigozy *et al.*, 1997). However, in DCs, uptake of both mycobacteria as well as of the glycolipids is performed mainly by DC-SIGN, leaving a minor role for the MMR (Schaible *et al.*, 2000; Geijtenbeek *et al.*, 2003; Driessen *et al.*, 2009;). Binding of Man-LAM to the MMR has been further linked to the delay of phagosome maturation (described in more detail in the corresponding section above) (Kang *et al.*, 2005), the induction of MMP-9 by *M. tuberculosis* and Man-LAM similar to that observed for TLR2 activation by lipomannan (Rivera-Marrero *et al.*, 2002) and the expansion of regulatory T cells (Tregs) (Garg *et al.*, 2008). Furthermore, DCs from MMR knockout mice secrete more IL-12p40 upon infection with *M. tuberculosis* as compared with DCs from wild-type mice (Ehlers, 2009). Thus, the MMR seems to have an immunosuppressive function on DCs, which was earlier shown by cross-linking the MMR on DCs with anti-MMR antibodies and several natural ligands (Chieppa *et al.*, 2003). Finally, in Mϕ, Man-LAM suppresses lipopolysaccharide-induced IL-12p40 secretion via the induction of IL-1 receptor-associated kinase (IRAK)-M expression (Pathak *et al.*, 2005). IRAK-M negatively regulates TLR signaling (Kobayashi *et al.*, 2002; Jo *et al.*, 2007;) and its induction is probably triggered by binding of Man-LAM to the mannose receptor (Pathak *et al.*, 2005).

Concluding, the MMR signaling may induce an anti-inflammatory response. A study by Torrelles *et al.* (2008b) reported, for clinical isolates of *M. tuberculosis* (HN885 and HN1554) with both truncated Man-LAM and a reduced amount of higher-order PIMs, a significant decrease in association with the MMR as compared with the Erdman strain, while uptake via the CR was not altered. Phagocytosis via the MMR may shape the nature of the immune response, and it was suggested that due to its immunosuppressive activity, uptake via the MMR may lead to a latent infection instead of active disease (Torrelles *et al.*, 2008b). Related to this, an *M. tuberculosis pimB*-mutant strain (Rv0557/*mgtA*), which expresses less lipomannan and Man-LAM in its cell wall, showed an increased rate of macrophage death upon infection as compared with its parent strain (Torrelles *et al.*, 2009). This does not necessarily mean increased virulence, but may even be advantageous for the host as the more virulent strains have been reported to inhibit apoptosis (Torrelles *et al.*, 2009). Although the *pimB*-mutant phenotype could not be linked to reduced association with the MMR, the presence of high amounts of PIMs, lipomannan and Man-LAM results in a less inflammatory immune response and indicates adaptation of the mycobacteria to their host (Torrelles *et al.*, 2009; Torrelles & Schlesinger, 2010;) (Fig. 7).

## Concluding remarks

The entire repertoire of enzymes and genes involved in the biogenesis of lipoarabinomannan and related glycoconjugates has almost been identified. However, unlike *C. glutamicum*, many of the genes involved in biosynthesis of these molecules in *M. tuberculosis* are essential for its survival (Sassetti *et al.*, 2003; Kaur *et al.*, 2009; Guerin *et al.*, 2010; G.S. Besra, unpublished data) like its cousin *M. smegmatis* (Jackson *et al.*, 2000; Kordulakova *et al.*, 2002; Guerin *et al.*, 2009; Skovierova *et al.*, 2009;), and therefore represent excellent drug targets. Furthermore, the identification of enzymatic machinery is now leading to the biophysical analysis of the three-dimensional structures of these enzymes and the identification of inhibitors, which may develop into novel drugs against tuberculosis. In addition, very little is known about the regulation of the biosynthesis of these glycoconjugates and their respective presentation on the mycobacterial cell wall; such studies may lead to the identification of few more interesting and potential targets. To date, serine–threonine kinases have been suggested in the regulation process of mycobacterial glycosyltransferases (Alderwick *et al.*, 2006b; Molle & Kremer, 2010;). However, further studies are required to unravel the detailed mechanism of regulation in terms of the species, composition, heterogeneity, size and length of these polymers.

PIMs, lipomannan and Man-LAM all display several immunomodulatory properties by interaction with different receptors of the immune system. Their activity depends on their degree of acylation and mannosylation. While lipomannan is mainly associated with TLR signalling, the higher-order PIMs and Man-LAM are recognized by the C-type lectins DC-SIGN and the MMR. Interestingly, alveolar Mϕ constitutively express the MMR (Wileman *et al.*, 1986), but DC-SIGN expression is only induced in alveolar Mϕ in the lungs of tuberculosis-infected patients (Tailleux *et al.*, 2005), which may influence the disease pattern in an important, but yet to be determined way. Both C-type lectins show comparable recognition specificities for the mannosylated glycolipids, and as receptors CD14 (Pugin *et al.*, 1994) and pulmonary surfactant protein (SP)-A (Sidobre *et al.*, 2000) and SP-D (Ferguson *et al.*, 1999, 2006) also bind Man-LAM, the interactions of mycobacteria with all these PRRs may enhance or dampen inflammatory signals and thereby determine the nature of the immune response (Ernst, 1998; Torrelles *et al.*, 2008a;). Anti-inflammatory signaling via the interaction of the glycolipids with the host immune system can be regarded as strategies of the mycobacteria to escape immune surveillance (Torrelles & Schlesinger, 2010), but may also be vital in the prevention of an exaggerated inflammatory response (Jo, 2008). As only 5–10% of the tuberculosis-infected persons develop the active disease, the glycolipids may play a crucial role in host adaptation of the mycobacteria and hence, a balanced immune response resulting in a latent tuberculosis infection (Fig. 7).

## Statement

Re-use of this article is permitted in accordance with the Terms and Conditions set out at http://wileyonlinelibrary.com/onlineopen#OnlineOpen-Terms
